# Contrasting marine carbonate systems in two fjords in British Columbia, Canada: Seawater buffering capacity and the response to anthropogenic CO_2_ invasion

**DOI:** 10.1371/journal.pone.0238432

**Published:** 2020-09-03

**Authors:** Alex Hare, Wiley Evans, Katie Pocock, Carrie Weekes, Iria Gimenez

**Affiliations:** 1 Hakai Institute, Heriot Bay, British Columbia, Canada; 2 Department of Zoology, University of British Columbia, Vancouver, British Columbia, Canada; Pacific Northwest National Laboratory, UNITED STATES

## Abstract

The carbonate system in two contrasting fjords, Rivers Inlet and Bute Inlet, on the coast of British Columbia, Canada, was evaluated to characterize the mechanisms driving carbonate chemistry dynamics and assess the impact of anthropogenic carbon. Differences in the character of deep water exchange between these fjords were inferred from their degree of exposure to continental shelf water and their salinity relationships with total alkalinity and total dissolved inorganic carbon, which determined seawater buffering capacity. Seawater buffering capacity differed between fjords and resulted in distinct carbonate system characteristics with implications on calcium carbonate saturation states and sensitivity to increasing anthropogenic carbon inputs. Saturation states of both aragonite and calcite mineral phases of calcium carbonate were seasonally at or below saturation throughout the entire water column in Bute Inlet, while only aragonite was seasonally under-saturated in portions of the water column in Rivers Inlet. The mean annual saturation states of aragonite in Rivers Inlet and calcite in Bute Inlet deep water layers have declined to below saturation within the last several decades due to anthropogenic carbon accumulation, and similar declines to undersaturation are projected in their surface layers as anthropogenic carbon continues to accumulate. This study demonstrates that the degree of fjord water exposure to open shelf water influences the uptake and sensitivity to anthropogenic carbon through processes affecting seawater buffering capacity, and that reduced uptake but greater sensitivity occurs where distance to ocean source waters and freshwater dilution are greater.

## 1. Introduction

Relatively little information exists on marine carbonate system dynamics within fjords along the Pacific coast of North America. The few studies from Prince William Sound [1], Glacier Bay [2], and Puget Sound [3, 4], describe these settings as highly variable with strong seasonality in carbonate system parameters and sensitive to ‘anthropogenic carbon’ (TCO_2_Anth_) addition resulting from the ocean uptake of rising atmospheric carbon dioxide (CO_2_). These characteristics are driven primarily by freshwater inputs, seasonal primary production, organic matter respiration at depth, and wind-influenced circulation patterns. The key role of freshwater has also been identified in fjords adjoining other ocean basins where it may directly impact the carbonate system by changing total dissolved inorganic carbon (TCO_2_), total alkalinity (TA), and carbonate (CO_3_^2-^) concentrations [5, 6], or by changing the air-sea carbon dioxide disequilibrium [7–9]. Cold freshwater inputs can also influence the carbonate system through additional thermodynamic effects resulting from seawater dilution that enhance undersaturated conditions with respect to the partial pressure of CO_2_ (pCO_2_) [8, 10]. High-latitude fjords in other oceans may also contain sea ice that enhances atmospheric CO_2_ uptake and transport to deeper waters through additional mechanisms such as brine formation and sea ice melt [11]. Consequently, the source and character of freshwater is important in these settings; for instance, cold glacial melt is commonly recognized as a major driver of atmospheric CO_2_ uptake in fjord surface waters due to low pCO_2_ content, [5, 8–10], and may simultaneously be undersaturated with respect to calcium carbonate (CaCO_3_).

Fjord surface seawater conditions are thus commonly corrosive for the key CaCO_3_-based mineral aragonite used in shell formation by a variety of aquatic calcifiers. Aragonite precipitation is thermodynamically favored when the aragonite saturation state (Ω_Ar_) is > 1, whereas dissolution is favored at Ω_Ar_ < 1, as determined by:

$$\Omega_{Ar}=[Ca^{2+}][CO_{3}{}^{2‐}]/K_{sp\left(arag\right)}$$ Eq 1

In Eq 1, Ca^2+^ is calcium, and K_sp(arag)_ is the mineral-specific solubility product and is temperature, salinity, and pressure-dependent; this term is ~1.5 times lower for the less soluble calcite phase of CaCO_3_ for which saturation, Ω_Ca_, is defined equivalently as for aragonite [12]. Thus, when seawater conditions are undersaturated with respect to calcite, both forms of calcium carbonate have a tendency to dissolve.

Increasing seawater uptake of TCO_2_Anth_ increases pCO_2_ and reduces seawater pH, CO_3_^2-^ concentrations, and CaCO_3_ mineral saturation states (Ω), processes collectively termed ocean acidification (OA). The extent of OA is mediated by seawater buffering capacity, which is determined by the ratio of TA to TCO_2_ and controls the carbonate system response to changes in TCO_2_ from either natural or anthropogenic sources. These relationships are described by the Revelle factor for pCO_2_ and by similar buffer factors for pH and Ω [13]. Revelle factor represents the fractional change in pCO_2_ per unit change in TCO_2_, such that weakly buffered seawater has a higher Revelle factor and demonstrates larger changes in pCO_2_, pH, and Ω relative to well buffered seawater for a given change in TCO_2_ [14]. Buffering capacity is typically lower in the North Pacific than at lower latitudes [13], and fjords in this region typically show lower Ω than both North Pacific surface water and fjord waters of other ocean basins (e.g. [1, 9, 15, 16]).

Ω levels are strong indicators of shell-forming conditions for marine calcifiers such as corals, coccolithophores, pteropods, and bivalves [17, 18], and are directly connected to their development, growth, and habitat quality [19–21]. Calcite has been examined less frequently in OA studies because the more soluble aragonite phase is the first to dissolve under increasingly acidic conditions, and has therefore been a main focus of investigations into biological responses to OA in, for instance, tropical corals, pteropods, and early larval stages of mollusks (e.g. [18, 22–24]). However, biogenic calcite is also susceptible to OA [25], and is widely used by many globally important species such as foraminifera and coccolithophores [26], and by locally important species along the Pacific coast such as adult oysters, mussels, and sea urchins [27, 28]. The north Pacific Ocean is also a region where the calcite saturation horizon, the depth at which Ω_Ca_ = 1, seasonally shoals to ~ 250 m in winter, and Ω_Ca_ declines along the continental shelf during summer periods of coastal upwelling [29–31]. It is therefore critical for coastal carbonate studies to document both Ω_Ca_ and Ω_Ar_ levels in regions where Ω_Ca_ is near saturation because the trajectory of OA implies that it will become an additional stressor to marine life in these regions in the future [32].

In this study, we examined the marine carbonate system in two contrasting fjords to evaluate how shelf water exposure influences their carbonate system patterns and uptake and response to TCO_2_Anth_. A 2.5-year long record of carbonate system and standard oceanographic measurements demonstrates how differences in the exchange and dilution of deep fjord water drive differences in carbonate system parameters and their response to TCO_2_Anth_. Furthermore, we estimate how the carbonate system parameters may change over the remainder of the century under the high CO_2_ emissions scenario associated with IPCC Representative Concentration Pathway (RCP) 8.5 [33].

## 2. Materials and methods

### 2.1 Study locations

**Fig 1:**
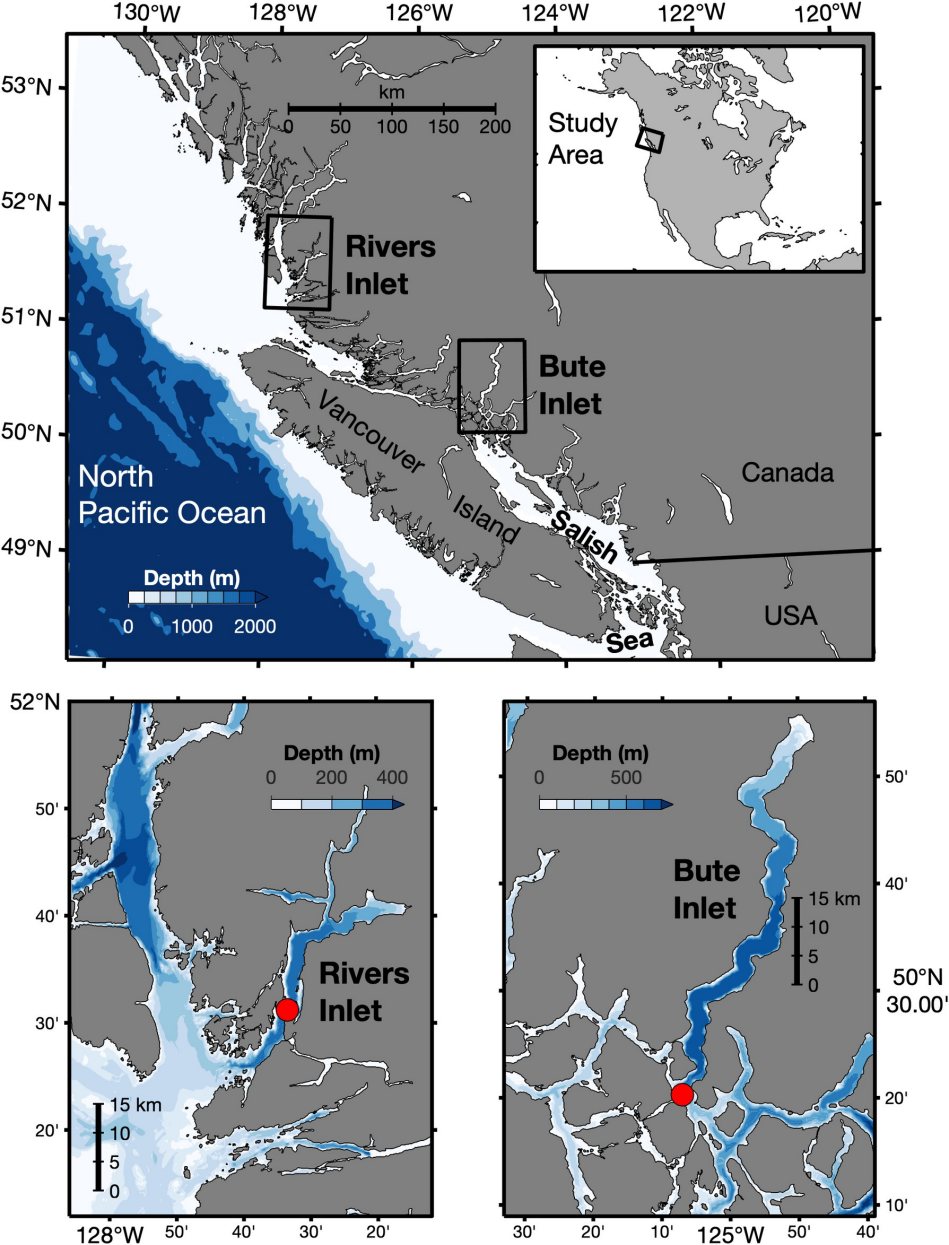
Regional and local maps of the study area and of Bute and Rivers inlets. Red circles indicate the sampling location in each fjord, located at 127.5583°W, 51.5208°N in Rivers Inlet and at 125.1176°W, 50.3392°N in Bute Inlet. Map contains bathymetric data from [40] and coastline data from [41].

Rivers Inlet (‘Rivers’) and Bute Inlet (‘Bute’) are long (45 km and 80 km, respectively) and deep (365 m and 650 m, respectively) fjords located on the coast of British Columbia (B.C.), Canada (Fig 1) [34, 35]. Bute is isolated from open continental shelf water to the southeast by ~400 km through the Salish Sea with which the majority of water exchange occurs over a relatively deep sill of ~265 m in intermediate channels [34, 36, 37], and roughly half that distance to the northwest from which additional minor water exchange occurs (Fig 1). In contrast, Rivers has a direct connection to the continental shelf and exchanges water over a broad and shallow sill ~135 m deep at its mouth. Major rivers present at the heads of both inlets (Rivers: Wannock River, Bute: Homathko River) deliver regionally significant freshwater inputs from rainfall, snow melt, and glacial melt [38], and drive estuarine-type circulation characterized by a shallow low-salinity surface layer (typically < 5–10 m) flowing seaward and a sub-surface return flow of higher-salinity water beneath [35, 37, 39]. Surface and deep water residence times in Rivers are ~ 1–2 weeks and ~ 6–11 months respectively, depending on river discharge rate and seasonal shelf upwelling [39]. Surface water residence time in Bute is ~ 2–4 weeks and no estimate is available for deep water [39], however, the residence time of sub-surface water in the adjoining Salish Sea is ≤ 1–3 years [36]. The freshwater content of sub-surface water differs greatly between the two systems, and Bute deep water salinity remains well below that of deep shelf water year-round, whereas Rivers deep water salinity seasonally matches that of deep shelf water.

### 2.2 Sample collection and analyses

Measurements are reported for one station in each fjord, located about midway along the fjord thalweg in Rivers and near the mouth of Bute (Fig 1). Bottle sampling (pCO_2_, TCO_2_, nutrients) using Niskin bottles (General Oceanics, Miami, U.S.A.) occurred in Bute from May 3, 2016 to January 17, 2019, and in Rivers from June 13, 2016 to September 14, 2018. Bottle samples were typically collected at 12 depths at each station at bi-weekly or monthly intervals, excluding several data gaps during winter months (December to February), particularly during the 2016/2017 winter in Rivers. We refer to March to May as spring, June to August as summer, and September to November as autumn. Water column and maximum sampling depths at each station were 320 m and 300 m in Rivers, and 550 m and 500 m in Bute. We refer to measurements from the deepest bottle sample collected as ‘bottom water’ for each fjord.

Ancillary hydrographic data (temperature, salinity, pressure, dissolved oxygen) were collected using a 19Plus V2 conductivity-temperature-depth (CTD; Sea-Bird Scientific, Bellevue, U.S.A.) profiler, or a Maestro RBR CTD profiler (RBR, Ottawa, Canada). CTD measurements were collected independently and alongside bottle samples at up to weekly intervals with no data gap greater than 1 calendar month during the time series. CTDs and ancillary sensors were serviced annually by their manufacturers, and sensor measurements were processed using standard protocols recommended by Sea-Bird Scientific and RBR. Calculations involving SI units (e.g. density) were performed using TEOS-10 equations [42], otherwise Practical Salinity (S_P_) is reported. Phosphate and silicate concentrations were determined from seawater filtered through a 0.45 μm filter, frozen and stored in the dark until analyzed at the University of British Columbia using a Lachat QuikChem 8500 Series 2 Flow Injection Analysis System and following conventional protocols [43, 44]. Nutrient analytical uncertainty as percent standard deviation was < 1% for all nutrients. pCO_2_ and TCO_2_ samples were collected in 350 mL amber glass bottles and preserved with 200 μl of saturated mercuric chloride solution following established CO_2_ sampling methods [45]. pCO_2_ and TCO_2_ samples were analyzed at the Hakai Institute’s Quadra Island Field Station using non-dispersive infrared CO_2_ absorbance with a gas analyzer (LI-COR LI840A CO_2_/H_2_O) following methods described elsewhere [46]. Accuracy and linearity for both parameters was ensured with the use of independently calibrated internal gas and liquid standards, and external Certified Reference Materials (CRM) from A.G. Dickson, Scripts Institution of Oceanography. CRMs were analyzed routinely during TCO_2_ analysis and demonstrated an accuracy of ± 0.3%. Field sampling uncertainty of bottle samples was also assessed by triplicate sample collection at one depth on each sampling date; mean standard deviation of replicate TCO_2_ measurements was 7.5 μmol kg^-1^. Estimated pCO_2_ uncertainty was ≤ 1.5% based on comparison of a known CRM TA value and that computed from measured TCO_2_ and pCO_2_ on that CRM. Uncertainty in the derived Ω and pH parameters determined by this methodology was ± 0.023 and ± 0.008 [46]. Sample data was also quality controlled by root mean squared error-based analyses of TA relationships with salinity as described in detail in S1 Text. Carbonate system parameters that were not directly measured were calculated using a MATLAB program for CO2SYS version 1.1 [47] with pCO_2_, TCO_2_, temperature, salinity, pressure [48], and silicate and phosphate concentrations as input variables. We used H_2_CO_3_ dissociation constants applicable for low salinity surface water in the fjords [49], the recommended sulfuric acid dissociation constant and borate ratio for CO2SYS computations [50, 51], and report pH on the total scale. Computed alkalinity is the ‘inorganic’ contribution, which includes CO_3_^2-^, HCO_3_^-^, borate and hydroxide ions, as well as H^+^, phosphate and silicate, and equals TA when organic acid concentrations are negligible, which at times may not be the case for surface water in close proximity to freshwater inputs in these systems [52]. The presence of organic alkalinity in river water at commonly reported concentrations elsewhere (e.g. [48, 53]) could potentially increase Ω by < 0.2 from values reported here, with greatest effect at low salinities (e.g. S_P_ < 10) present in a limited number of surface samples (S1 Fig). The potential effect on pH in low-salinity samples is appreciably larger (S1 Fig) and we refrain from interpreting low-salinity pH measurements. River discharge data was obtained from the Water Survey of Canada [54]. Rivers Inlet field sampling included access via the Hakai/Luxvbalis Conservancy under B.C. Parks permits # 107070 and # 107090, while Bute Inlet field sampling required no access through any marine park, preserve, or otherwise restricted area. Field studies did not involve endangered or protected species and did not require special permissions for seawater collection or ancillary hydrographic data collection.

### 2.3 Net community production

Monthly Net Community Production (NCP) was calculated as the difference in monthly mean salinity-normalized TCO_2_ concentrations (ΔnTCO_2_), corrected for changes in salinity-normalized alkalinity (ΔnTA) and NO_3_^-^ uptake (ΔNO_3_^-^), between samples collected in consecutive months, according to Eq 2 [55, 56].

$$\Delta nTCO_{2}=([nTCO_{2,Month\left(1\right)}]–[nTCO_{2,Month\left(2\right)}])‐(\Delta [nTA]+\Delta [NO_{3}{}^{‐}]\times 0.5),\mu mol\;kg^{‐1}$$ Eq 2

**Fig 2:**
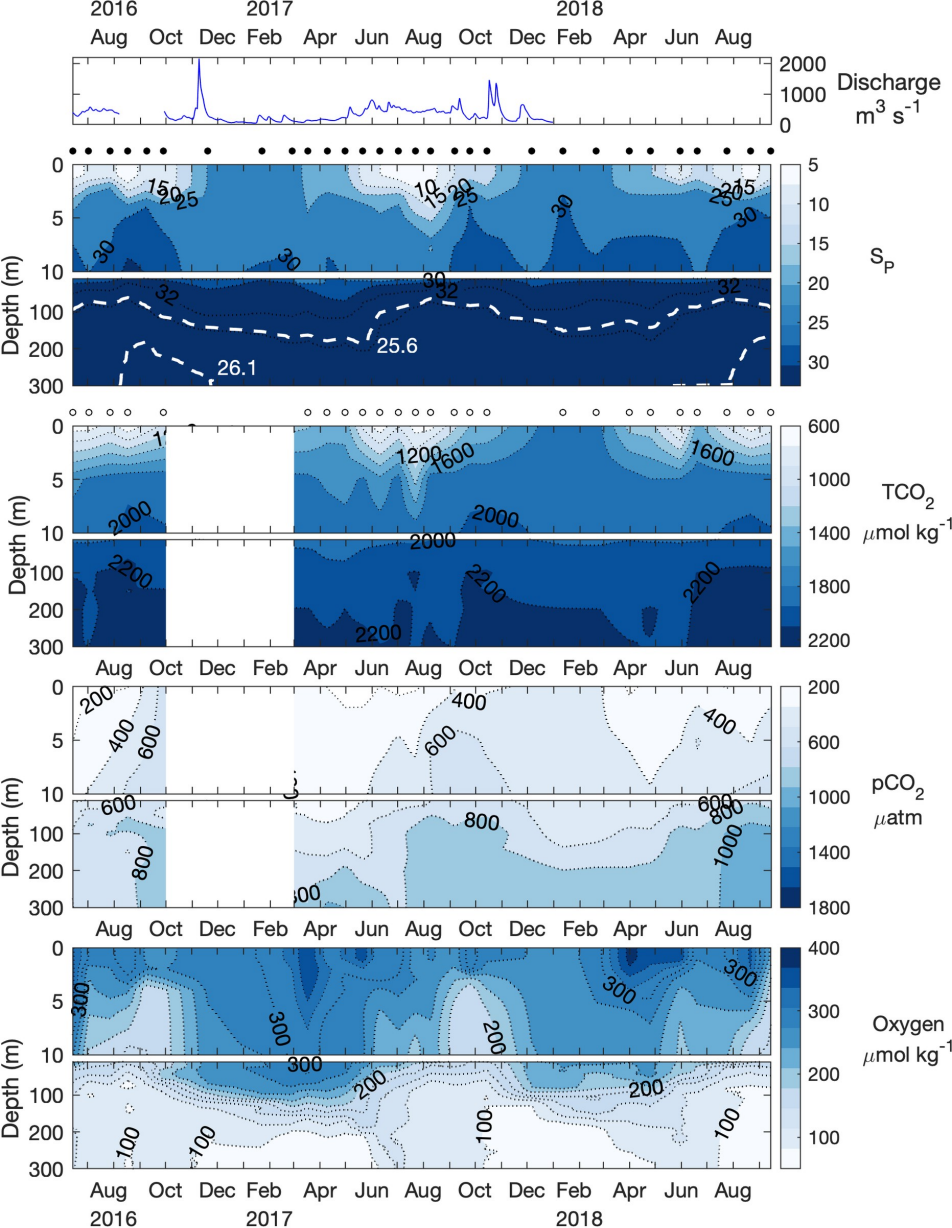
Time series of river discharge, salinity, TCO_2_, pCO_2_, and oxygen in Rivers Inlet. Black circles between panels indicate CTD sampling dates, white circles indicate bottle sampling dates. White dashed lines in the salinity panel represents isopycnals for σ_θ_ = 25.6 kg m^-3^ and σ_θ_ = 26.1 kg m^-3^, respectively, that approximate mid and peak seawater density in the Rivers Inlet deep layer. Colorbar scales match those in Fig 3.

**Fig 3:**
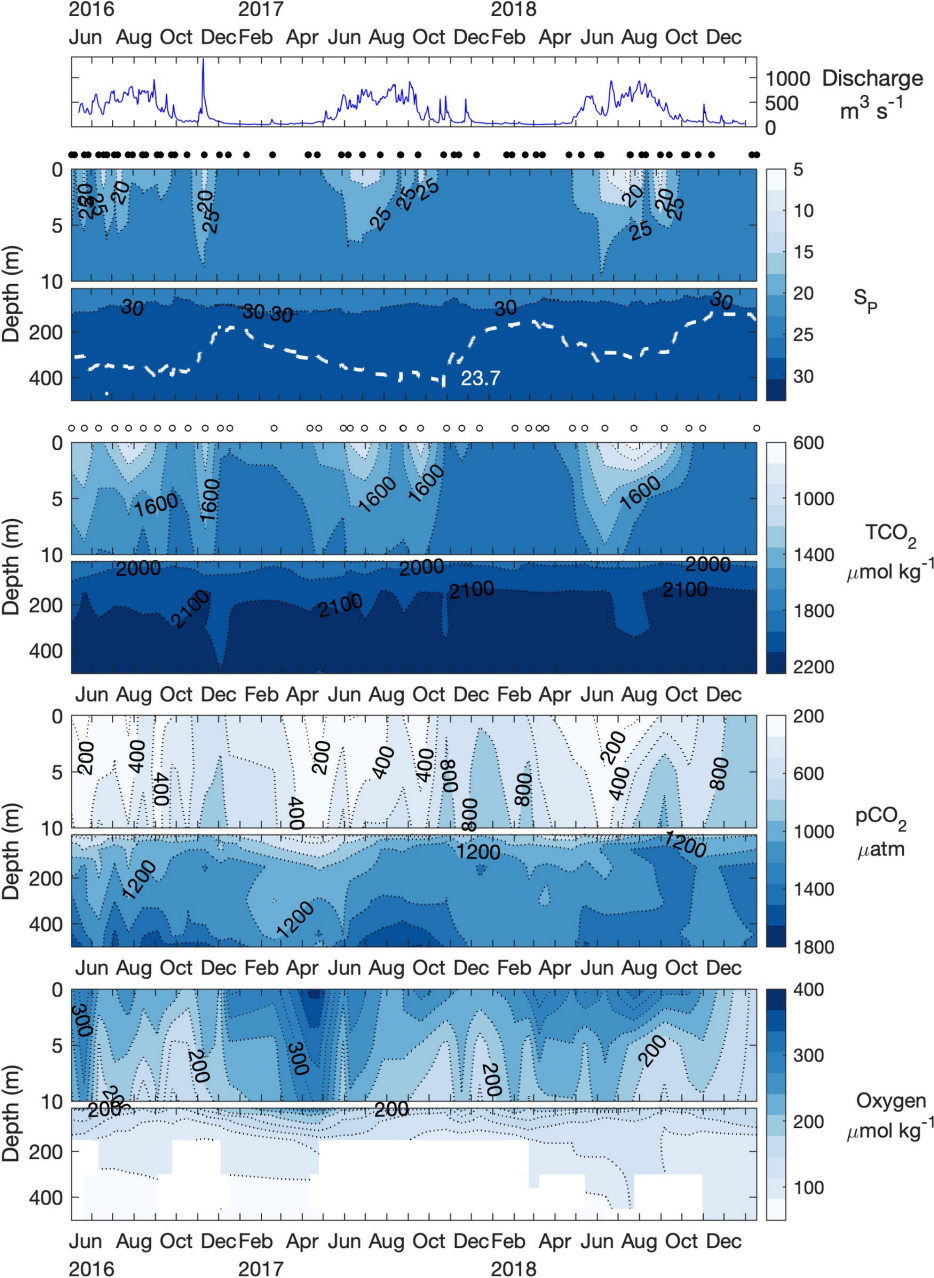
Time series of river discharge, salinity, TCO_2_, pCO_2_, and oxygen in Bute Inlet. Black circles between panels indicate CTD sampling dates, white circles indicate bottle sampling dates. White dashed line in the lower salinity panel represents the isopycnal for σ_θ_ = 23.7 kg m^-3^ that approximates peak seawater density in Bute Inlet. Colorbar scales match those in Fig 2.

Monthly mean TCO_2_ was determined by averaging measurements at each depth sampled within the upper 30 m of the water column during each month, which encompassed the majority of undersaturated pCO_2_ measurements (e.g. Figs 2 and 3), and then averaging depth-means within each calendar month. This approach minimized depth-bias where sample size for a given depth was unequal between months and averaged inter-annual variability in the pCO_2_ undersaturation depth. nTCO_2_ and nTA were computed using Eq 3 to account for non-zero freshwater end-member concentrations.

$$nTCO_{2}=([TCO_{2,measured}]–[TCO_{2,}{}_{Sp=0}])/S_{P}\times nS+[TCO_{2,Sp=0}],\;\mu mol\;kg^{‐1}$$ Eq 3

TCO_2,Sp = 0_, (or TA_Sp = 0_) is the TCO_2_ (or TA) concentration at S_P_ = 0 and was determined empirically from all samples within the selected layer for each system separately, and nS is the reference salinity of 35 [57].

Inventories of corrected ΔnTCO_2_ were then produced by multiplying this term by the mean seawater density (kg m^-3^) between sampling periods, and by the integration depth in meters. Carbon production or loss rates were determined by dividing inventory changes by 30 days which approximated the mean time interval between months, and reported as mmol C m^-2^ d^-1^ with positive values representing net organic carbon production (autotrophy) and negative values representing net organic carbon loss (heterotrophy). Uncertainties reported for the above calculation represent the cumulative contribution from standard error in the mean monthly TCO_2_ concentrations and analytical uncertainties in TCO_2_, TA, and nutrient measurements.

### 2.4 Anthropogenic carbon

The accumulation of TCO_2_Anth_ and its effects on water properties were estimated with the ΔTCO_2_ method [58], which is an adaptation of the ΔC* method [59]. The methods assume that seasonal variation in seawater TA, salinity, temperature, and the disequilibrium of seawater TCO_2_ (ΔTCO_2_) remain consistent over the time periods investigated, and estimate TCO_2_Anth_ with uncertainty of ≈ 10% [60]. ΔTCO_2_ is the difference between measured seawater TCO_2_ concentrations (TCO_2_measured_) and those corresponding to seawater at equilibrium with current TA and atmospheric pCO_2_ (TCO_2_modern_equilibriated_), and is determined by Eq 4:

$$\Delta [TCO_{2}]=[TCO_{2\_measured}]‐[TCO_{2\_modern\_equilibrated}],\;\mu mol\;kg^{‐1}$$ Eq 4

Estimates of TCO_2_ concentrations for other years (TCO_2_year_) were determined by subtracting ΔTCO_2_ from TCO_2_ concentrations corresponding to seawater with modern TA and at equilibrium with the atmospheric pCO_2_ for that year (TCO_2_year_equilibrated_) using Eq 5:

$$[TCO_{2\_year}]=\Delta [TCO_{2}]‐[TCO_{2\_year\_equilibrated}],\;\mu mol\;kg^{‐1}$$ Eq 5

Atmospheric pCO_2_ levels for the years 1765 to 2100 were determined by converting CO_2_ mole fractions associated with the RCP 8.5 scenario [33] to partial pressure assuming a constant 1 atm of pressure. TCO_2_year_ estimates were then used with modern TA, temperature, and salinity to derive the remaining parameters of the carbonate system for those years. TCO_2_Anth_ for a given year was determined as the difference between TCO_2_year_ and TCO_2_1765_. TCO_2_, TA, and pCO_2_ measurements from the year 2017 were used as modern values because the greatest sampling coverage occurred during this year in both fjords. In addition to assumptions outlined above, this approach is sensitive to the age-estimation of the water mass being evaluated [61]. Seawater age measurements are not available in either fjord but were approximated by oxygen utilization rates as described in section 3.4 below. Seawater TCO_2_Anth_ content was estimated for water in recent contact with the atmosphere and the deep layer separately to account for the relative ‘age’ of water in each layer, which relates to the atmospheric pCO_2_ at their time of last atmospheric contact. Given the trajectory of atmospheric pCO_2_, sub-surface water would have experienced lower atmospheric pCO_2_ than modern surface water and therefore contains lower TCO_2_Anth_ content (e.g. [62, 63]) We considered the surface layer to vertically mix sufficiently to interact with current atmospheric pCO_2_ (Figs 2 and 3).

## 3. Results

### 3.1 Water column structure

The water column at both fjord stations was characterized by distinct surface and deep water layers that were separated by an intermediate mixing layer that varied in depth seasonally and between fjords but is demarcated here by the top of the halocline at ~5 m depth, and the sill depth (Figs 2 and 3). The surface layer was most evident in both fjords from spring to autumn and demonstrated large seasonal salinity and temperature ranges, with lowest salinity and peak temperatures between June to August in both locations. The surface layer depth was less clearly defined in Bute than in Rivers, and periods of reduced salinity (< 27.5) and warmer temperatures (> 12°C) were intermittently measured to 10 m depth each summer, and occasionally to 20 m (Fig 3, S2 Fig). Mean salinity during summer months was lower and more variable in Rivers (18.9 ± 9.9) than in Bute (23.4 ± 3) but the mean temperature difference was < 1 degree (Rivers: 12.5 ± 2.9°C vs. Bute: 13.3 ± 1.6°C) (S2 Fig). The vertical gradient between the surface and intermediate layers in both fjords was reduced during autumn and winter and surface layer salinity and temperature became similar to that of upper intermediate water.

The intermediate water layer displayed smaller vertical gradients and magnitudes of seasonal change, with roughly opposite seasonal patterns from those observed in the surface layer (Figs 2 and 3). Rivers intermediate layer salinity increased from spring to summer, peaked in late summer or early autumn, and declined through winter. Seasonality of the Bute intermediate layer was similar to that of Rivers but delayed in timing as salinity peaked between winter and the following spring. The seasonality of intermediate layer temperature also lagged surface layer temperature changes in both locations, with winter cooling persisting until summer, and summer warming persisting into early winter (S1 Fig). This lag was strongest in late 2016 following summertime intermediate layer temperatures that were 0.4 to 0.6°C higher than in following years, which may reflect the 2015/2016 El Niño or residual warming from a marine heatwave that preceded it in the north Pacific [64].

Temperature and salinity in the deep layers of both fjords showed similar seasonality as observed in intermediate layers (Figs 2 and 3, S1 Fig). Mean deep layer salinity in Rivers (33.3 ± 0.1) was higher and peaked in late summer compared to Bute (30.7 ± 0.1), which peaked in early winter. Mean deep layer temperature was somewhat lower in Rivers (7.4 ± 0.2°C) than in Bute (9.3 ± 0.2°C).

### 3.2 Seasonal carbonate system patterns

#### 3.2.1 TCO_2_

**Fig 4:**
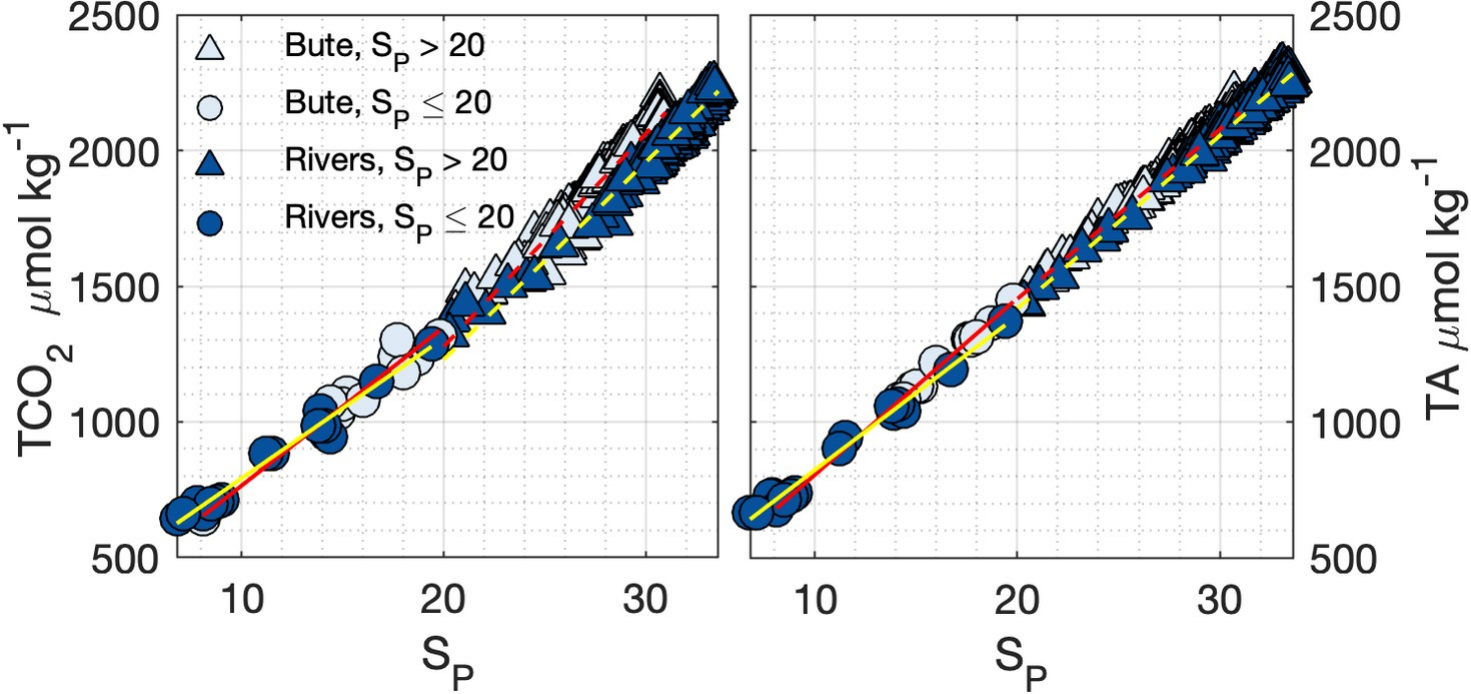
TCO_2_ and TA relationships with salinity in Rivers and Bute inlets. Solid and dashed lines indicate least-squares regressions for samples with salinity ≤ 20 (circles) and > 20 (triangles), respectively, for Rivers (yellow lines) and Bute (red lines). Regression parameters are listed in S1 Table.

TCO_2_ concentrations tracked seasonal salinity patterns at all depths in both fjords, although this pattern was less consistent in Bute deep water (Figs 2 and 3). Surface layer TCO_2_ concentrations reflected the seasonality of major river discharges to fjord headwaters, typically declining during spring and summer when river discharge was high, and increasing during periods of low river discharge (Figs 2 and 3). TCO_2_ reached minima of ~640 μmol kg^-1^ at salinity ≤ 8 during summer and generally increased in autumn to > 1900 μmol kg^-1^ by winter alongside surface layer salinities > 29 in both fjords (Figs 2 and 3). A noticeable exception to this seasonality occurred during November 2016 when the surface TCO_2_ concentration in Bute was reduced to < 1500 μmol kg^-3^ during a period of very high river discharge (Fig 3). Intermediate layer TCO_2_ concentrations generally remained above peak surface layer concentrations except during periods of lowered TCO_2_ (< 1800 μmol kg^-1^) that frequently extended to ≥ 10 m depth during summer and autumn. The seasonality of sub-surface layer TCO_2_ was reduced and contrasted with the surface layer by increasing over summer and declining during winter (Figs 2 and 3). This pattern was weaker in Bute where peak annual TCO_2_ concentrations in sub-surface layers typically occurred 1–2 months later compared to those in Rivers (Figs 2 and 3). Deep layer TCO_2_ concentrations were generally > 2200 μmol kg^-1^ in Rivers and peaked during periods of highest annual salinity, but were somewhat lower in Bute at 2100–2200 μmol kg^-1^ and tracked salinity less clearly. For example, the lowest deep layer TCO_2_ concentrations in Bute in 2016 occurred in December simultaneously with highest annual salinity, and TCO_2_ did not reflect subsequently declining salinity throughout the following summer (Fig 3). Despite enriched TCO_2_ relative to salinity in Bute (Fig 4A), TCO_2_ concentrations were generally lower than at equivalent depths in Rivers because salinity was lower in the former fjord (Figs 2 and 3).

#### 3.2.2 pCO_2_

pCO_2_ demonstrated similar seasonality and vertical distribution as TCO_2_ but was not strongly related to salinity, instead demonstrating an inverse correlation with oxygen (r^2^ > 0.81, p < 0.001). Annual surface layer minima preceded those of TCO_2_ by 1–2 months and typically occurred with high oxygen saturation levels. Surface layer pCO_2_ declined to 120 μatm to 165 μatm between March and May in Rivers and to 115 μatm to 148 μatm between April and June in Bute, when oxygen saturation reached 120% (364 μmol kg^-1^) to 130% (383 μmol kg^-1^), respectively (Figs 2 and 3). Surface layer pCO_2_ was continually undersaturated with respect to the atmosphere each year from March to September in Rivers and from April to July in Bute. The depth and magnitude of pCO_2_ undersaturation progressively declined throughout summer, however, surface pCO_2_ (i.e., 0 m samples) briefly returned to undersaturated conditions each year between late August and mid-October in Bute. pCO_2_ undersaturation also occurred to > 10 m depth on several occasions in both fjords, most significantly during spring 2017 when pCO_2_ undersaturation reached 50 m for ~1 month in Rivers Inlet (Fig 2). Surface layer pCO_2_ was considerably higher in Bute than in Rivers except during undersaturated periods, typically reaching ~450 μatm in Rivers and > 700 μatm in Bute during winter. The seasonality of sub-surface layer pCO_2_ contrasted with the surface layer and peaked during spring and summer and declined over winter (Figs 2 and 3). Deep layer pCO_2_ peaked first in April in Rivers and in August in Bute, which coincided with the period of lowest annual deep layer salinity in both fjords. A late summer peak followed in both deep and intermediate layers in Rivers that coincided with the period of highest annual sub-surface layer salinity, but this association was not readily apparent in Bute. The deep layer spring peak represented the highest pCO_2_ (1050 μatm) and lowest oxygen concentrations (87 μmol kg^-1^) in 2017, but the late summer peak had higher pCO_2_ (1160 μatm) and lower oxygen (79 μmol kg^-1^) concentrations in 2018. Sub-surface layer pCO_2_ was substantially higher in Bute at > 1800 μatm compared to ~1160 μatm in Rivers.

#### 3.2.3 Derived parameters

**Fig 5:**
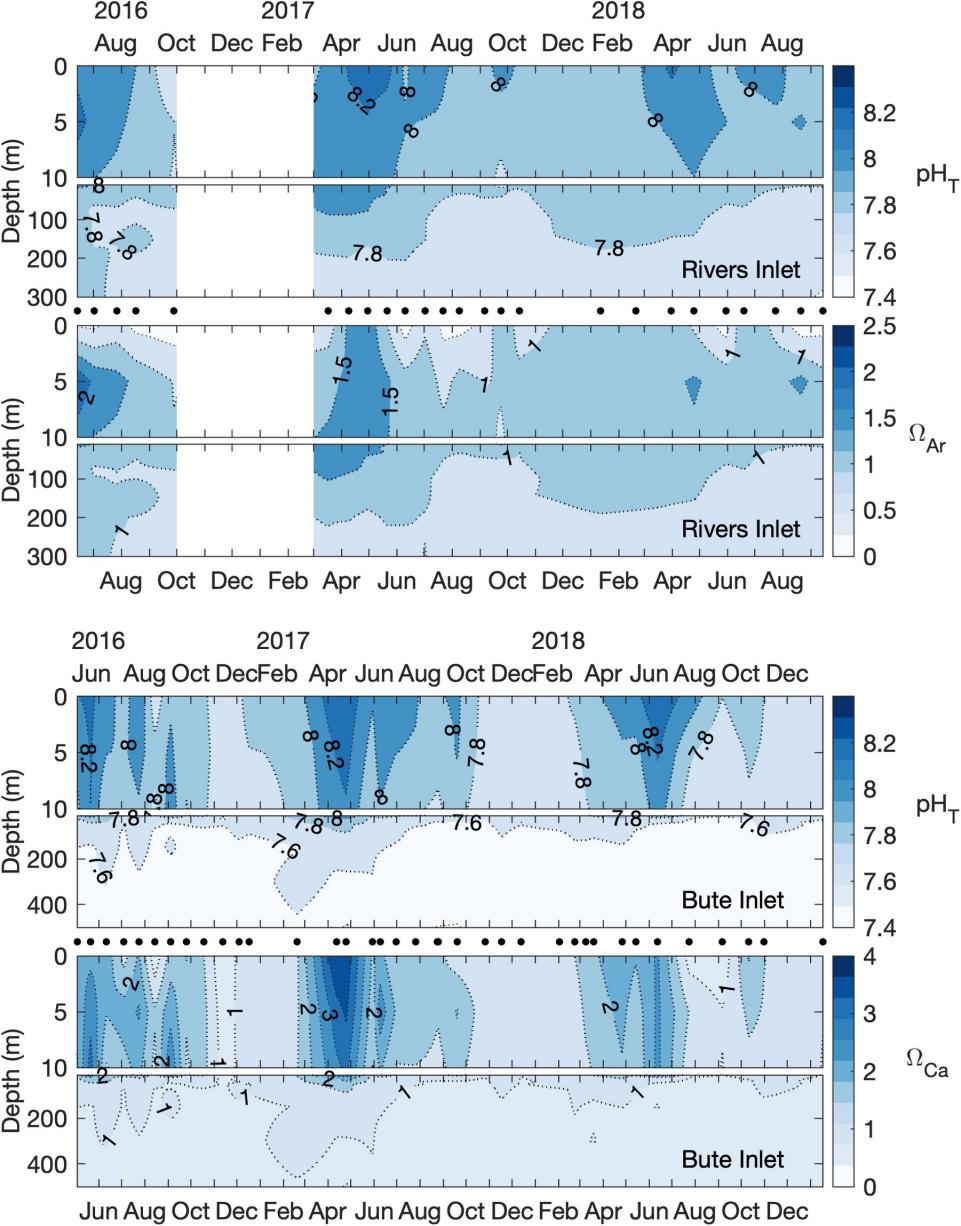
**Time series of pH and Ω**_**Ar**_ **in Rivers Inlet (top panels), and pH and Ω**_**Ca**_ **in Bute Inlet (bottom panels).** Black circles between panels indicate sampling dates. Contours of 1 in the lower Ω panels represent the corresponding saturation horizon depth. Note different y-axis scales between Ω_Ar_ and Ω_Ca_ panels.

The derived carbonate system parameters TA, pH, Ω_Ar_, and Ω_Ca_ also exhibited differences between fjords and enhanced seasonal variability in the surface layer relative to deeper layers. TA concentrations showed nearly identical linear relationships with salinity between both fjords but were consistently lower in Bute in sub-surface layers because salinity was lower than in Rivers (Fig 4B, S3 Fig). Seasonal TA patterns mirrored TCO_2_ and salinity (S3 Fig) while pH, Ω_Ar_, and Ω_Ca_ patterns were inversely correlated with pCO_2_ except during periods of very low salinity when Ω levels decoupled from this relationship (e.g. August 2017, Rivers and Bute, and August 2018, Bute, Fig 5, S3 Fig). Surface layer pH seasonality was similar between fjords and reached 8.3 during spring and generally declined towards winter but remained ≥ 7.8 in Rivers and ≥ 7.6 in Bute (Fig 5). pH also declined with depth and reached annual minima of 7.6 and 7.4 in Rivers and Bute deep layers, respectively. Surface layer Ω_Ar_ and Ω_Ca_ tracked pH patterns and peaked in spring and declined over summer and with increasing depth in both fjords (Fig 5). Peak Ω_Ar_ and Ω_Ca_ reached 2.4 and 4.0, respectively, in the surface layer during the study period, and were typically higher at 5 m depth during summer than at the surface. Surface Ω levels were frequently well below saturation (0.2 to 0.6) during summer periods of low salinity and were occasionally below minimum levels observed in the deep layers. The aragonite saturation horizon (excluding low-salinity surface measurements) shoaled through summer to between 10 m and 50 m depth in Rivers and to the surface in Bute by late summer, then deepened to below sill depth during winter in Rivers while aragonite remained undersaturated throughout the water column for up to 4.5 months during autumn and winter in Bute (Fig 5, S4 Fig). In contrast, calcite remained saturated throughout the study period in Rivers with the exception of brief low-surface salinity periods in summer, but a calcite saturation horizon was present in Bute that shoaled to between 30 m to 40 m by late summer (Fig 5, S4 Fig). Calcite remained saturated in the surface and upper intermediate layers in Bute except during a brief period in late fall 2016 that coincided with an unusually high discharge event from the Homathko River, during which time calcite was undersaturated throughout the water column (Fig 3).

#### 3.2.4 Seawater buffering capacity in Rivers and Bute inlets

Seawater buffering capacity was lower in Bute compared to Rivers because of greater freshwater dilution and enriched seawater TCO_2_ concentrations relative to salinity. TCO_2_ concentrations in both fjords were linearly related to salinity (r^2^ ≥ 0.97, p < 0.001, S1 Table) but were higher in Bute than in Rivers for a given salinity above approximately S_P_ = 20, and nearly identical with slightly lower slopes at lower salinity (Fig 4A). The freshwater TA/TCO_2_ ratios implied by these relationships also indicated that dilution further decreased the seawater buffering capacity in Bute relative to Rivers (S1 Table). The mean sub-surface water TA/TCO_2_ ratios in Bute and Rivers were 1.01 and 1.04, respectively, which correspond to a thermodynamic equilibrium CO_2_ concentration ~54% greater in Bute (1017 μatm) than in Rivers (661 μatm) at the mean sub-surface conditions in each fjord, which reflected the actual mean sub-surface layer pCO_2_ (Bute: 1060 μatm, Rivers: 692 μatm).

### 3.3 Net community production

**Fig 6:**
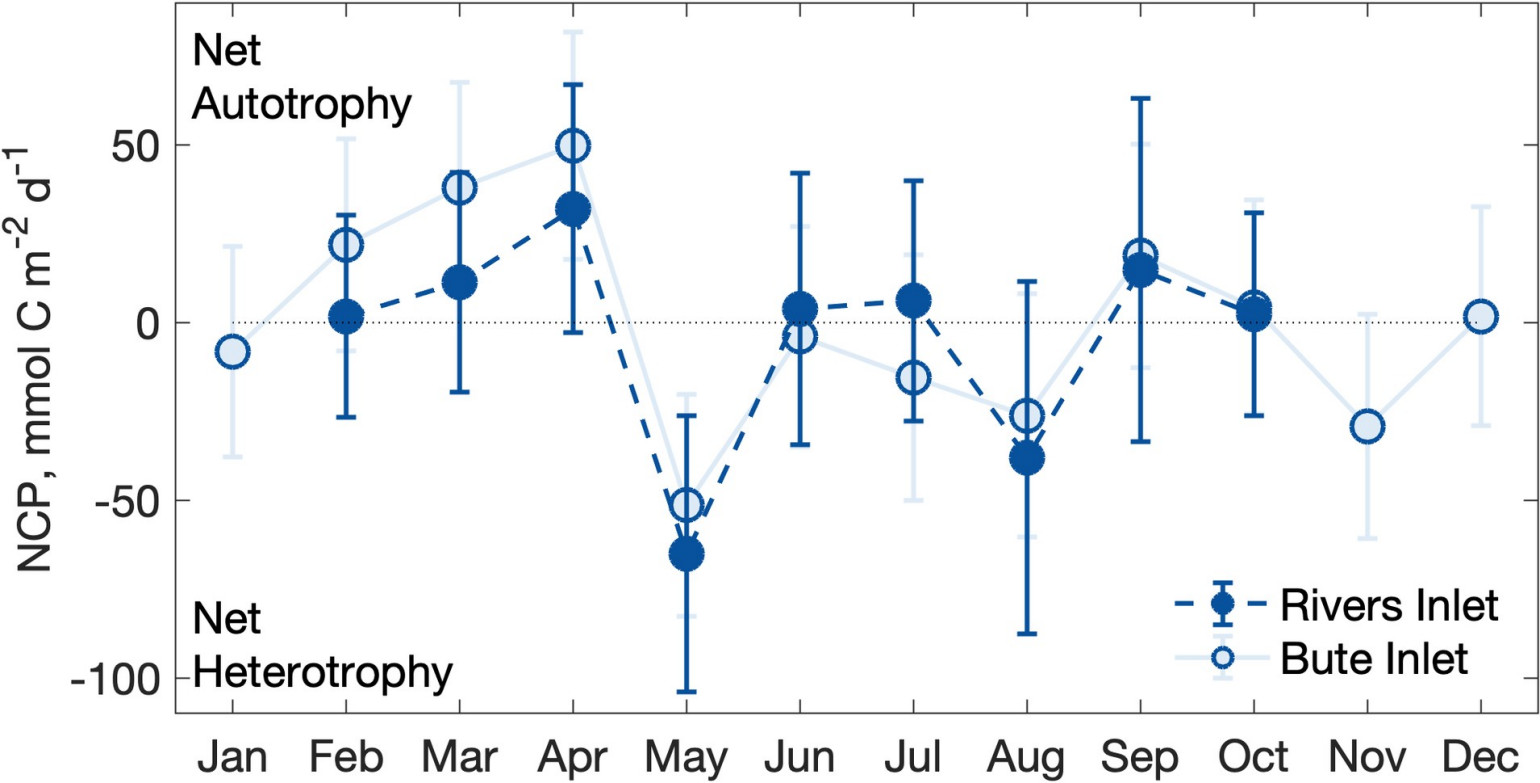
Mean monthly NCP (mmol C m^-2^ d^-1^) in the upper 30 m of Rivers and Bute inlets. Error bars represent standard error of the mean TCO_2_ concentration in each month and the analytical uncertainties in TCO_2_, TA, and nutrient samples.

The mean annual NCP cycle in both fjords implied a near-balance between autotrophic and heterotrophic conditions in most months due to large variability between years (Fig 6). Mean monthly NCP was net autotrophic beginning in February and peaked in April (Rivers: 32 ± 35 mmol C m^2^ d^-1^_,_ Bute: 50 ± 32 mmol C m^2^ d^-1^), followed by peak net heterotrophy in May (Rivers: -65 ± 39 mmol C m^2^ d^-1^, Bute: -51 ± 31 mmol C m^2^ d^-1^) (Fig 6, S2 Table). Mean monthly NCP was near or below zero during the summer months and returned to low net autotrophy in September in both fjords. Although the integrated annual NCP was net heterotrophic in Rivers at -30.8 ± 113 mmol C m^2^ d^-1^ and nearly balanced in Bute at 0.2 ± 109 mmol C m^2^ d^-1^, these estimates contained large uncertainties because monthly means demonstrated considerable variability between years (Fig 6).

### 3.4 Anthropogenic carbon (TCO_2_Anth_) content

Estimated TCO_2_Anth_ in the surface layer was 50 ± 4 μmol kg^-1^ in Rivers and 47 ± 3 μmol kg^-1^ in Bute, for the year 2017 (Table 1). Estimated TCO_2_Anth_ in the deep layers was lower, reflecting a greater seawater age and lower atmospheric pCO_2_ at the time of last atmospheric contact. Since deep water residence times appear < 1–3 years in both fjords [36, 39], most ‘aging’ occurs outside the fjords in the Pacific Ocean where these waters originate. Therefore, we estimated deep water age as the quotient of the apparent oxygen utilization (AOU, μmol kg^-1^) and oxygen utilization rates (OUR, μmol kg^-1^) as determined elsewhere in the north Pacific (e.g. [65]). We neglected the relatively minor residence time for sub-surface water within the Salish Sea [66] and the effect of incoming water mixing in Haro Strait because this process has a negligible effect on TCO_2_ concentrations due to rapid transit times and slow CO_2_ gas exchange kinetics [67]. Although Haro Strait mixing increases oxygen content of incoming water to the Salish Sea [67], deep water (> 250 m) oxygen concentrations in the northern Salish Sea were limited to a narrow concentration range (< 50 μmol kg^-1^) between April and December [68], implying that such mixing has less impact on deep water oxygen at the northern end of the strait. Bottom water AOU was 194 ± 15 μmol kg^-1^ and 195 ± 14 μmol kg^-1^ in Rivers and Bute, respectively, and using the mean OUR of ~5 μmol kg^-1^ yr^-1^ estimated for north Pacific water between 200 and 500 m depths [65] produces maximum deep water ages of 39 ± 3 years in both fjords. The corresponding estimated TCO_2_Anth_ for the deep layer was 34 ± 3 μmol kg^-1^ and 32 ± 2 μmol kg^-1^ for Rivers and Bute, respectively (Table 1). TCO_2_Anth_ estimates are sensitive to seawater age estimates; if the estimated age of California Undercurrent water off the Washington state coast, a potential source of fjord deep water, is used (25 years, [69]), deep water TCO_2_Anth_ increases to 41 μmol kg^-1^ and 38 μmol kg^-1^ in Rivers and Bute, respectively. TCO_2_Anth_ estimates are also sensitive to projected atmospheric pCO_2_ levels, but estimated current TCO_2_Anth_ was robust to the moderately lower CO_2_ levels associated with the RCP 6.0 scenario [70]; surface and deep layer TCO_2_Anth_ content was ≤ 2 μmol kg^-1^ lower under these conditions (S3 Table).

**Table 1:** Estimates of mean^a^ annual TCO_2_Anth_, pH, and Ω by water layer for selected years.

Parameter	Year	Rivers Inlet		Bute Inlet	
		Surface	Deep	Surface	Deep
**TCO**_**2_Anth**_	1765	0	0	0	0
μmol kg^-1^	2017	49.5 ± 3.5	33.8 ± 3.4	47.2 ± 2.9	32.3 ± 1.6
	2100	139.1 ± 9.1	124.1 ± 7.2	132.7 ± 7.9	117.6 ± 4.8
**pH**	1765	8.11 ± 0.14	7.85 ± 0.08	8.12 ± 0.17	7.64 ± 0.06
	2017	7.96 ± 0.16	7.75 ± 0.07	7.97 ± 0.19	7.52 ± 0.07
	2100	7.63 ± 0.19	7.44 ± 0.06	7.65 ± 0.24	7.24 ± 0.06
	Δ_2017_^b^	-0.16 ± 0.21	-0.11 ± 0.11	-0.15 ± 0.25	-0.12 ± 0.09
	Δ_2100_^c^	-0.48 ± 0.24	-0.41 ± 0.10	-0.47 ± 0.29	-0.38 ± 0.08
**Ω**_**Ca**_	1765	2.75 ± 0.55	1.86 ± 0.33	2.75 ± 0.9	1.11 ± 0.17
	2017	2.06 ± 0.52	1.49 ± 0.25	2.08 ± 0.83	0.86 ± 0.14
	2100	1.05 ± 0.35	0.76 ± 0.12	1.1 ± 0.59	0.46 ± 0.06
	Δ_2017_	-0.69 ± 0.76	-0.37 ± 0.41	-0.67 ± 1.22	-0.25 ± 0.22
	Δ_2100_	-1.7 ± 0.65	-1.1 ± 0.35	-1.65 ± 1.01	-0.65 ± 0.18
**Ω**_**Ar**_	1765	1.72 ± 0.34	1.18 ± 0.21	1.70 ± 0.56	0.70 ± 0.11
	2017	1.29 ± 0.32	0.94 ± 0.16	1.29 ± 0.52	0.54 ± 0.09
	2100	0.66 ± 0.21	0.48 ± 0.07	0.68 ± 0.37	0.29 ± 0.04
	Δ_2017_	-0.43 ± 0.47	-0.24 ± 0.26	-0.41 ± 0.76	-0.16 ± 0.14
	Δ_2100_	-1.06 ± 0.40	-0.70 ± 0.22	-1.02 ± 0.67	-0.41 ± 0.12

^a^ Mean ± standard deviation

^b^ Δ_2017_ = Change in mean value from the year 1765 to the year 2017

^c^ Δ_2100_ = Change in mean value from the year 1765 to the year 2100

**Fig 7:**
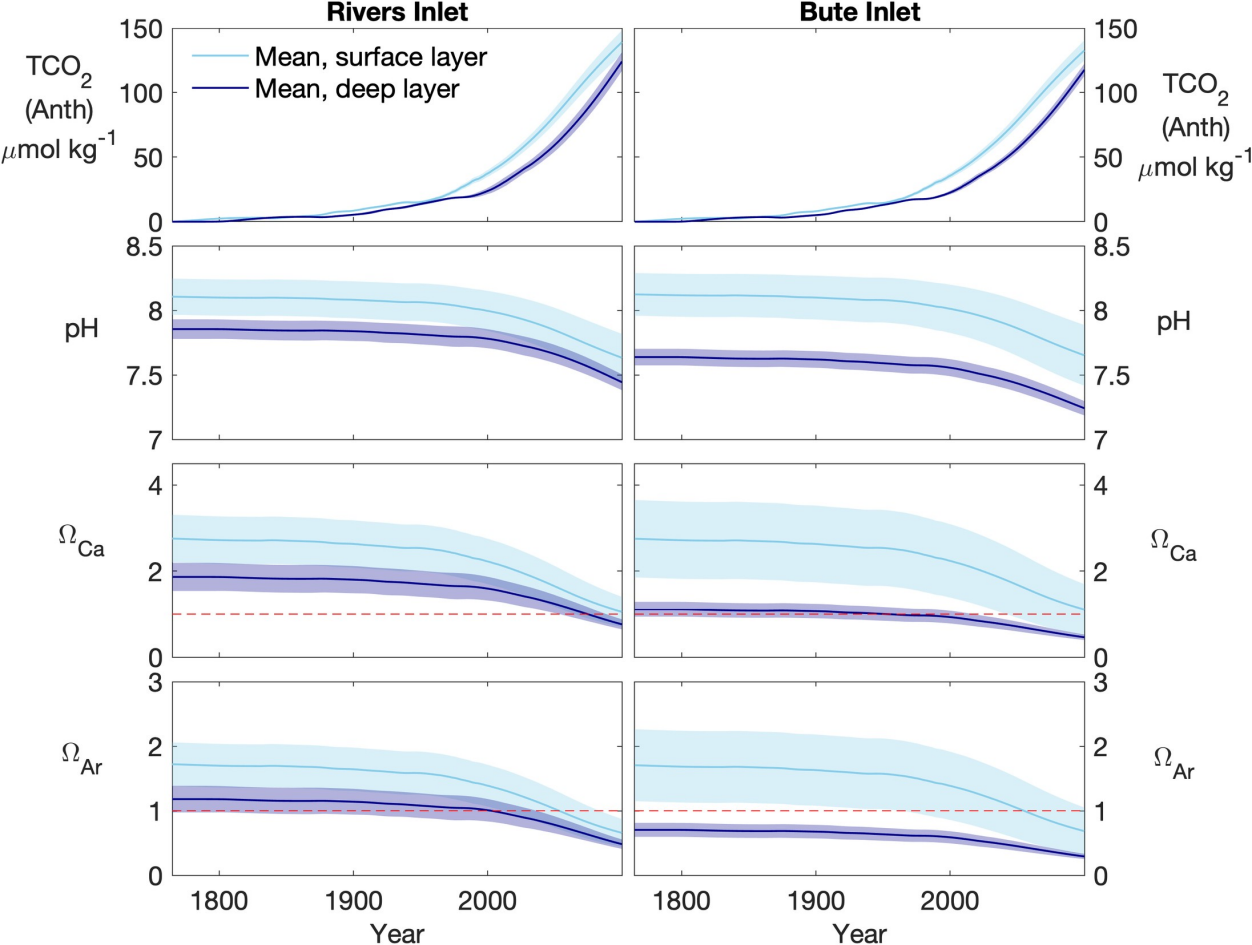
Trajectories of TCO_2_Anth_, pH, Ω_Ca_, and Ω_Ar_ determined in Rivers and Bute inlets for the years 1765 to 2100 under atmospheric pCO_2_ conditions associated with the RCP 8.5 scenario. Solid lines and shaded regions represent annual means and standard deviations, respectively, for surface (light blue) and deep (dark blue) water layers. Red dashed lines in lower 4 panels indicate the corrosivity threshold (i.e., Ω = 1).

The estimated current and projected accumulation of TCO_2_Anth_ in Bute and Rivers inlets indicates relatively low accumulation during the first half of the industrialized era and more rapidly increasing TCO_2_Anth_ accumulation after the years ~1950 in the surface layer and ~2000 in the deep layer (Fig 7). TCO_2_Anth_ accumulation is also reflected by declining pH and Ω levels, with annual variability slightly increasing in surface layer pH and decreasing in Ω under higher TCO_2_Anth_ conditions. These changes are projected to continue to the year 2100 under the atmospheric pCO_2_ projection associated with the RCP 8.5 scenario (Fig 7, Table 1). Mean annual pH has declined from preindustrial conditions to the year 2017 by -0.16 to -0.15 in in Rivers and Bute surface layers, respectively, and by -0.11 to -0.12 in their deep layers (Table 1). These current changes in mean annual pH are projected to roughly triple by the year 2100. Similar patterns emerged for Ω, for which surface layer Ω_Ca_ declined by -0.69 and -0.67 in Rivers and Bute, respectively, and in their deep layers by -0.37 and -0.25, by the year 2017. Ω_Ar_ declined in parallel to Ω_Ca_ at roughly 61% to 65% of the magnitude observed for Ω_Ca_. These trends indicated that the deep layer mean annual Ω declined from supersaturation prior to the presence of TCO_2_Anth_ to undersaturation, for Ω_Ar_ in Rivers and Ω_Ca_ in Bute, by the years 2003 ± 5 and 1953 ± 7, respectively, considering 10% TCO_2_Anth_ uncertainty (Fig 7). Further declines are projected forwards and estimated surface layer mean annual Ω_Ar_ is < 1 by the year 2056 ± 8 in both fjords, and only Ω_Ca_ remains > 1 in the surface layer in either location by the year 2100. The timing of crossing these thresholds is also sensitive to the atmospheric pCO_2_ levels, and surface layer mean annual Ω_Ar_ remains above saturation until the year 2074 ± 11 in both fjords under pCO_2_ conditions corresponding to the lower emissions of the RCP 6.0 scenario (S3 Table).

## 4. Discussion

### 4.1 The governing role of seawater buffering capacity

**Fig 8:**
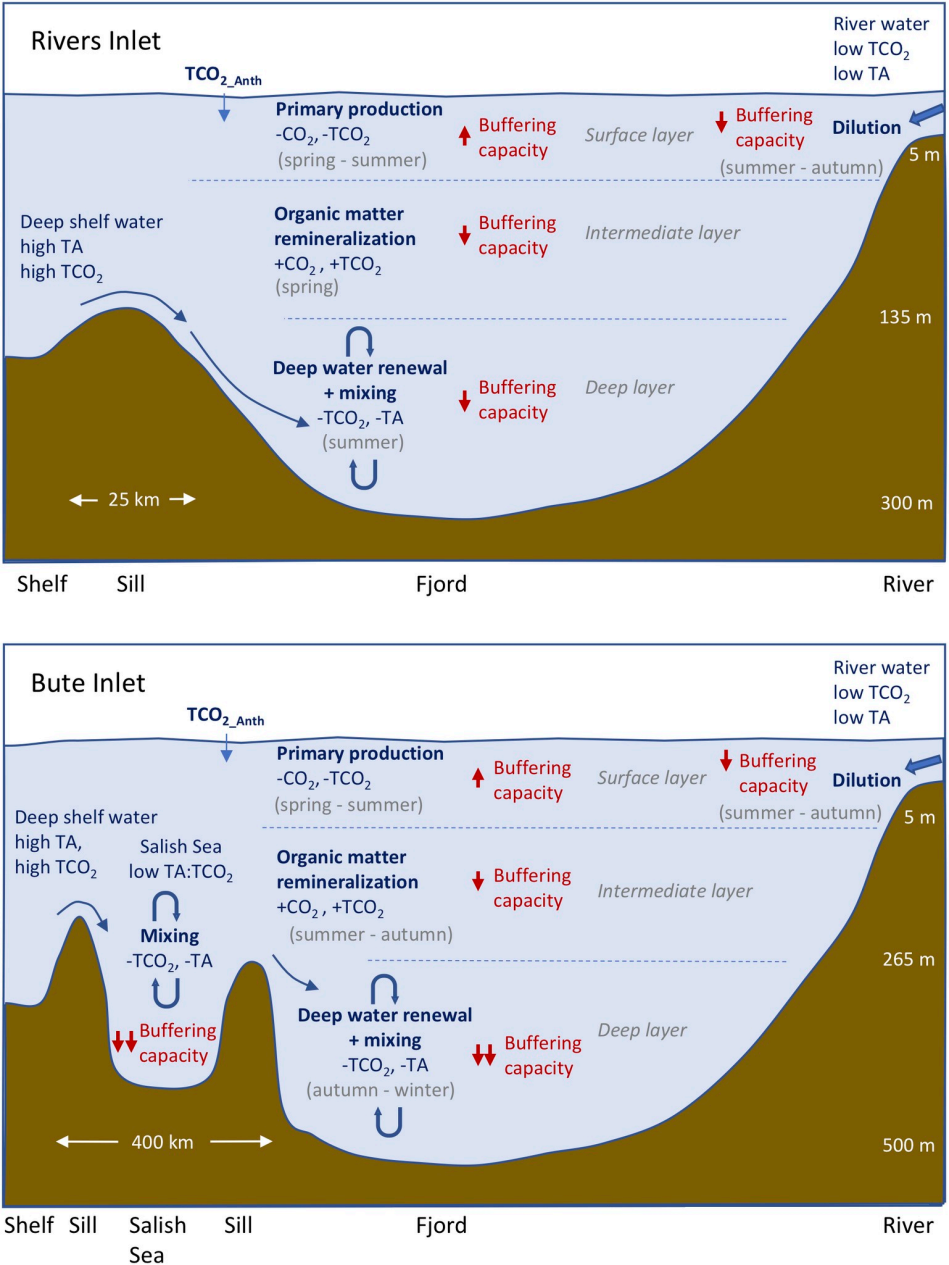
Conceptual diagram of the major processes controlling buffering capacity in Rivers and Bute inlets. Increasing levels of buffering capacity and carbonate system parameters are indicated by upwards arrows and addition (+) signs, respectively, decreasing levels are indicated by downwards arrows and negative (-) signs. Processes are shown in bold text within water layers where they likely contribute greatest, but may also contribute in other layers. Seasonality of peak observed effects from the major processes are shown in nearby parentheses. Distances and depths are approximate, and not drawn to scale.

Differences between the carbonate systems of Rivers and Bute were primarily driven by different seawater buffering capacities that reflected their exposure to the continental shelf, with lesser contributions from biological processes, river discharge, and gas exchange (Fig 8). Incoming shelf water to both fjords contained higher buffering capacity that was subsequently lowered by mixing with brackish fjord water (Fig 8). This effect was greater in Bute seawater because incoming shelf water first mixes with brackish Salish Sea water that has enriched TCO_2_ content and lower buffering capacity relative to shelf waters of the same salinity [46, 67], and by marginally lower TA/TCO_2_ in freshwater discharge to Bute (Figs 4 and 8, S1 Table). The significance of deep water transit through the Salish Sea was evident in Bute deep layer salinity that was roughly 10% diluted from shelf water compared to < 2% in Rivers (Figs 2 and 3). Organic matter remineralization within the fjords also contributed to reduced seawater buffering capacity (Fig 8), and this process appeared more influential in Bute where low buffering capacity increased the effect of remineralization on pCO_2_ levels. The impact of lower relative buffering capacity in Bute was less apparent in the surface layer where primary productivity independently lowered pCO_2_ levels. The sensitivity of these systems to TCO_2_Anth_ was thus governed by their buffering capacity but modulated by local controls on carbonate system dynamics.

### 4.2 Control of surface carbonate system dynamics

Freshwater dilution had a clear effect on carbonate system dynamics because the greatest observed variability in TCO_2_ and TA was associated with large salinity changes that matched major river discharge patterns in both fjords (Figs 2 and 3). For instance, seasonal changes in surface layer TCO_2_ concentrations between consecutive winter and spring sampling dates declined by roughly six-fold when salinity-normalized (Figs 2 and 3, S2 Table). Freshwater dilution also produced undersaturated surface Ω conditions because low salinity water contained low CO_3_^2-^ concentrations irrespective of pCO_2_ levels (S6 Fig). Such decoupling between pCO_2_ and Ω has been observed previously in coastal settings where it was attributed to both low CO_3_^2-^ concentrations and cold temperatures of river discharge and sea ice and tidewater glacial melt [1, 71]. Freshwater Ca^2+^ concentrations that deviate from conservative seawater salinity relationships may also impact Ω values in low salinity waters [72]. Although freshwater Ca^2+^ concentrations were not directly measured in this study, peak Ca^2+^ concentrations recorded in both the Homathko and Wannock rivers (S4 Table) would be insufficient to compensate for the low CO_3_^2-^ concentrations in summer surface layer seawater and increase Ω to saturation. Freshwater can also reduce summertime pCO_2_ by lowering the thermodynamic equilibrium concentration of CO_2_ because this effect is most significant in low salinity mixtures of fresh and marine water (e.g. [8]). This possibility was assessed by using freshwater TCO_2_ and TA concentrations implied from their salinity relationships (Fig 4) and from highest salinities in each fjord, along with corresponding river water temperatures (S1 Fig, S4 Table), to compute corresponding pCO_2_ concentrations (pCO_2mix_) across the mixing gradient. The difference between pCO_2mix_ and pCO_2_ in equilibrium with the contemporary atmosphere was 23 μatm below saturation at the minimum salinity of Rivers samples, and above atmospheric saturation along the entire mixing gradient in Bute (S5 Fig). Consequently, freshwater mixing could account for ~11% of the observed undersaturation at the lowest salinity observed in Rivers, but could not account for undersaturation in Bute. This effect was also dependent on cold river temperatures because if higher river temperatures were used (e.g. 15°C), undersaturation of pCO_2mix_ in Rivers disappeared. The presence of organic alkalinity in river water could also increase the potential contribution of freshwater mixing to undersaturated pCO_2_ at low salinities (S5 Fig).

Although freshwater mixing could not drive significant atmospheric CO_2_ uptake as observed in fjords elsewhere [1, 8, 9], some atmospheric CO_2_ uptake presumably occurred during summer when surface pCO_2_ levels were undersaturated but increased towards autumn (Figs 2 and 3). Part of this increase can also be attributed to coinciding moderate increases in temperatures and mixing as stratification weakened towards autumn, which typically affect surface layer pCO_2_ more rapidly than air-sea CO_2_ equilibration [73]. Similarly, gas efflux to the atmosphere presumably occurred during supersaturated pCO_2_ winter conditions in both fjords but this process did not appreciably affect surface pCO_2_ levels because early and late winter conditions were similar.

Biological processes primarily affected surface layer pCO_2_, Ω, and pH conditions during spring and summer. These observations were most evident during periods of increased oxygen saturation that occurred without accompanying changes in solubility. For example, large changes in pCO_2_, Ω_Ar_ and Ω_Ca_ occurred in Bute from winter 2016 to spring 2017 as oxygen saturation increased by > 50% but oxygen solubility remained nearly constant. Biological drawdown of pCO_2_ presumably diminished by mid-summer because the pCO_2_ sea-air gradient was roughly half to one third that of springtime, only some of which was attributable to warmer water temperatures. This pattern was partly consistent with the mean annual NCP cycle that suggested primary production lowered surface water TCO_2_ during April, contributed TCO_2_ through respiration in May, but was not distinguishable from zero in summer (Fig 7). Since net heterotrophy during May was not reflected by the undersaturated surface water pCO_2_ and generally high surface pH at this time, ecosystem function, in terms of autotrophy and heterotrophy, may at times be decoupled from surface pCO_2_ saturation status in these fjords. This circumstance has been observed elsewhere when seasonal changes in freshwater and temperature produced undersaturated surface water pCO_2_ during periods of winter respiration [74]. In contrast to primary production, calcification and dissolution had little effect on surface layer carbonate system parameters because maximum seasonal changes in nTA for both fjords constituted a negligible fraction of mean surface layer nTCO_2_ concentrations during summer (S2 Table). Similarly, respiration within the surface layer was likely negligible because of limited delivery or retention of organic matter supply, and high surface pCO_2_ during winter reflected upwards mixing of deeper water (Figs 2 and 3).

Vertical mixing between water layers was a driver of surface layer carbonate system variability primarily during winter when strong winds are prevalent, and river discharge and stratification are reduced (Figs 2 and 3). The higher winter pCO_2_ and lower pH and Ω levels in Bute compared to Rivers reflected vertical mixing with more weakly-buffered sub-surface water in the former system. Although lateral advection could also influence the surface layer carbonate system, it is unlikely that the observed conditions reflected imported signals of processes elsewhere because both fjords exhibit strong estuarine circulation year-round [34, 75].

### 4.3 Control of sub-surface carbonate system dynamics

The relative exposure of fjord waters to the continental shelf was a major determinant of sub-surface layer carbonate system properties by determining the extent of shelf water dilution and thereby exerting the primary control on seawater buffering capacity (Fig 8). The pattern of deep water renewal in both fjords is associated with seasonal upwelling along the continental shelf that typically begins in early summer and terminates in late summer [76]. The presence of upwelled source water in Rivers was indicated by deep layer properties and seasonal variability that corresponded to conditions representative of deep shelf water (e.g., S_P_ ≥ 33, σ_θ_ = 26.1 kg m^-3^, [29, 64]) and matched the upwelling season (Fig 2). Sub-surface TCO_2_ and pCO_2_ concentrations in Rivers also tracked seasonal upwelling (Fig 2), and late summer bottom water measurements closely matched those in deep continental shelf break water or upwelled water elsewhere along the northeast Pacific coast [29, 77, 78]. Increasing deep layer pCO_2_ and oxygen in summer also reflected upwelled source water because in situ respiration would be unable to account for the observed increase given the low organic matter supply implied from NCP (Fig 6, [79]), and would otherwise reduce oxygen levels. However, these comparisons varied inter-annually; upwelled water in the summer of 2018 contained both the highest pCO_2_ and the lowest oxygen concentrations measured in Rivers during the study (Fig 2).

The influence of shelf water was less apparent in Bute because sub-surface water TCO_2_ concentrations did not show obvious seasonal peaks and pCO_2_ less consistently tracked the seasonal upwelling pattern (Fig 3). This discontinuity exists in part because deep water exchange in Bute occurs through the Salish Sea rather than directly with deep shelf water as in Rivers [80], thereby delaying its arrival and modifying its composition through internal processes. For instance, peak density (σ_θ_ = 23.7 kg m^-3^) in Bute was well below that characteristic of upwelled water, but typical of the heavily diluted deep water of the northern Salish Sea (Fig 3) [36, 80]. This signature was also reflected in Bute deep water TCO_2_ concentrations that were higher relative to salinity than in upwelled shelf water, but never reached as high concentrations as in shelf water because peak salinity was lower [67]. The longer source water pathway to Bute was also evident because the timing of changes in deep layer conditions lagged those observed in Rivers by several months and were consistent with the slow northward progression of deep water renewal through the Salish Sea [36]. Consequently, the time period between deep water exchange ended in late summer in Bute (Fig 3), and elevated deep layer pCO_2_ at this time reflected in situ respiration rather than incoming high-pCO_2_ shelf water as in Rivers. Bottom water pCO_2_ subsequently declined as deep water exchange occurred with lower-pCO_2_ water from the Salish Sea in late fall or early winter (Fig 3).

The contribution from in situ respiration to deep layer TCO_2_ content during the non-renewal period was estimated by using the difference in TCO_2_ content between incoming deep water and bottom water prior to deep water exchange (S5 Table), after accounting for dilution and freshwater TCO_2_ content (S1 Table). This method assumes that primary productivity, calcification, dissolution, and vertical exchange are negligible in the deep layer, and that significant advection is limited to the renewal periods. Although these assumptions are generally robust (e.g., S2 Table), declining deep water salinity and density during the non-upwelling period indicated that some mixing occurs between sub-surface layers and may reduce estimates using this method. The estimated contribution of in situ respiration to deep layer TCO_2_ concentrations is 24 μmol kg^-1^ in Rivers and 137 μmol kg^-1^ in Bute, using this approach. However, the Bute estimate includes TCO_2_ accumulated by shelf water during passage through the Salish Sea where respiration is a major process governing organic matter removal [81]. Replacing shelf water with northern Salish Sea deep water properties yields an estimated contribution of 67 μmol kg^-1^ TCO_2_ from in situ respiration in Bute. This estimate is consistent with an amplified response from organic matter respiration in its more weakly buffered waters compared to Rivers.

### 4.4 Implications of low buffering capacity on TCO_2_Anth_ content and response

A consequence of lower buffering capacity in Bute was lower TCO_2_Anth_ content but slightly increased variability in most conditions, which is projected to increase further by the end of the century (Table 1). This circumstance illustrates the role of buffering capacity in determining seawater TCO_2_Anth_ content, and demonstrates that system response may differ between closely located coastal settings as observed elsewhere across regional scales. For example, lower buffering capacity in the North Pacific also enhances carbonate chemistry sensitivity to TCO_2_Anth_ relative to the Gulf of Mexico [14]. Deep layer pCO_2_, pH and Ω conditions in Bute also resembled those of offshore water from below the continental shelf-break [29, 78], whereas deep layer conditions in Rivers generally resembled nearby shelf bottom water. Estimates of deep layer conditions in Bute prior to TCO_2_Anth_ uptake indicate naturally low pH, undersaturated Ω_Ar_, and Ω_Ca_ near saturation, whereas modern deep water conditions in Rivers that include TCO_2_Anth_ are less corrosive (i.e., higher pH, higher Ω) than those in the preindustrial Bute deep layer (Table 1, Fig 7). These conditions in Bute are unusual even compared to Alaskan fjords and the offshore northeast Pacific where Ω tends to be low relative to comparable systems elsewhere ([1, 2, 8, 9, 30]). For example, calcite saturation horizons can shoal to < 250 m during winter in the northeast Pacific [30], whereas the winter calcite saturation horizon in Bute shoaled to depths of ~20 m to 50 m. In combination with surface water undersaturation, this shoaling reduced Ω_Ca_ to ≤ 1 throughout the water column briefly during the study period (Fig 5). Such circumstances may represent extremes along the northeast Pacific coast but are not necessarily unique within the Salish Sea because low-salinity deep water with low buffering capacity is a feature of this region [3, 31, 48]. Equally low sub-surface pH (7.4) and Ω_Ar_ (< 0.5) were reported in a highly restricted portion of the southern Salish Sea with persistently low sub-surface salinity and low buffering capacity (i.e., TA/TCO_2_ < 1) [3].

Both fjords displayed lower TCO_2_Anth_ content relative to offshore waters, which was consistent with regional differences in buffering capacity and Revelle factor [13, 82]. For instance, estimated TCO_2_Anth_ in the surface layer (Table 1) was lower compared to offshore Pacific surface water (~65 μmol kg^-1^, [62, 63]) where Revelle factors are typically lower than in coastal regions [83]). Deep layer mean TCO_2_Anth_ (Table 1) was similarly below recent estimates for near-bottom shelf waters (39 μmol kg ^-1^, [84]) and the California Undercurrent off the Washington coast (36 μmol kg^-1^, [69]). Higher Revelle factors in coastal waters also imply increased sensitivity to TCO_2_Anth_ for other carbonate system parameters. This relationship was evident in the greater decline in surface layer pH (Table 1) than the global surface ocean average (-0.11, [82]) despite lower TCO_2_Anth_ content, and from slightly enhanced pH variability in Bute compared to Rivers that also reflected their different sensitivities to TCO_2_Anth_ accumulation (Fig 7) [13, 58].

The lower buffering capacity in Bute seawater could exacerbate physiological responses in species vulnerable to OA to a greater degree in this fjord by enhancing the impact of TCO_2_Anth_ on the periods of high-pCO_2,_ low-pH, and extensive CaCO_3_ undersaturation in the water column observed here (Fig 3). OA is associated with adverse physiological responses across a range of marine taxa including reduced survival, growth, calcification, suppressed metabolism, altered behavioral responses, and intensified sensitivities to multi-stressors including reduced oxygen and elevated temperature ([17, 18]). In many coastal settings, including Bute and Rivers, pCO_2_, pH and Ω show seasonal and spatial decoupling, and recent experimental work has shown different physiological sensitivities to these parameters [19, 85]. Such circumstances warrant particular attention in coastal settings where carbonate chemistry is highly dynamic and enhanced variability is projected under increasing TCO_2_Anth_ because sensitivity thresholds might be crossed earlier than in more well-buffered locations (e.g. [46, 58]).

## 5. Conclusions

This study demonstrated that the distinct carbonate system patterns in two fjords of the North Pacific are principally driven by differences in their exposure to shelf water, which impacts source water characteristics and largely determines fjord seawater buffering capacity. These differences have affected TCO_2_Anth_ uptake and response, with reduced uptake and more acute responses where seawater dilution and distance to ocean basin source waters are greater. Therefore, fjords without direct connections to the open continental shelf will likely demonstrate greater sensitivity to OA. Both fjords have currently accumulated sufficient TCO_2_Anth_ that the annual mean Ω_Ar_ in Rivers and Ω_Ca_ in Bute deep water layers have declined to below saturation within the last several decades. The continuing TCO_2_Anth_ accumulation projected to occur under the atmospheric CO_2_ trajectories associated with the RCP 6.0 and RCP 8.5 scenarios indicate that the mean annual Ω_Ar_ in the surface layer of both fjords could decline to below saturation before the end of the century. This study also demonstrated that coastal OA studies investigating biological impacts should evaluate declining Ω_Ca_ in addition to Ω_Ar_ where low seawater buffering capacity is present or expected to develop.

## Supporting information

S1 Text Quality assurance and control of bottle sample measurements. (DOCX)

S1 Fig Potential effects of organic alkalinity in river water on computed Ω and pH. Blue dashed lines represent difference in Ω_Ar_ (panels A, C) and pH (panels B, D) computed from estimated TA and TCO_2_ relationships (S1 Table) plus organic alkalinity at concentrations indicated in figure legend, and without additional organic alkalinity. Legend in panel D applies to all panels. Red vertical lines indicate minimum salinity of 0 m and 5 m samples. Red circles indicate salinity distribution of all samples for the corresponding fjord.
(TIF)

S2 Fig Time-series of temperature in Rivers and Bute inlets. Black circles above panels indicate sampling dates.
(TIF)

S3 Fig Time series of Total Alkalinity (TA) in Rivers and Bute inlets. Black circles above panels indicate sampling dates.
(TIF)

S4 Fig Time series of Ω_Ca_ in Rivers Inlet and Ω_Ar_ in Bute Inlet. Black circles above plots indicate sampling dates. Contours of 1 in the lower Ω_Ar_ panel represent the saturation horizon.
(TIF)

S5 Fig Measured and potential pCO_2_ undersaturation in Rivers and Bute inlets. ΔpCO_2_ represents atmospheric pCO_2_ subtracted from the pCO_2_ corresponding to estimated freshwater and seawater TA and TCO_2_ concentrations determined from seawater relationships and river properties (S1 and S4 Tables) for mixing ratios from 0 to 1, represented by salinity of the mixture, without (dashed lines) and with (dotted lines) 10 μmol kg^-1^ organic alkalinity (orgAlk). ΔpCO_2_ is computed similarly from measured pCO_2_ for surface (i.e., 0 m) samples (filled circles). Red dotted line indicates atmospheric saturation.
(TIF)

S6 Fig Selected relationships between carbonate system parameters and buffering capacity. Colorbar applies to all panels.
(TIF)

S1 Table Least-squares regression parameters for TCO_2_ and TA relationships with salinity in Bute and Rivers inlets. (DOCX)

S2 Table Mean Net Community Production (NCP) by month for the upper 30 m of the water column in Bute and Rivers inlets. (DOCX)

S3 Table Selected output conditions of the ΔTCO_2_ method under pCO_2_ trajectories associated with the RCP 8.5 and RCP 6.0 scenarios. (DOCX)

S4 Table Mean water temperature in the Homathko and Wannock rivers between May and August for the years 2017 to 2019. (DOCX)

S5 Table Bottom water properties used to estimate deep water respiration in Rivers and Bute inlets. (DOCX)
