# Supplementary material for: Contrasting marine carbonate systems in two fjords in British Columbia, Canada: Seawater buffering capacity and the response to anthropogenic CO2 invasion
Source: PLoS One. 2020 Sep 3;15(9):e0238432. doi: 10.1371/journal.pone.0238432 (PMC7470366; doi:10.1371/journal.pone.0238432)
Supplement: S1 Text — (DOCX) [file pone.0238432.s001.docx]

**S1 Text. Quality assurance and control of bottle sample measurements**

For each fjord dataset, bottle sample measurements were quality controlled by first examining for discrepancies in temperature and salinity relative to CTD measurements for these parameters at equivalent depths. Large deviations in salinity could indicate erroneous depth-targeting during sample collection, and large deviations in temperature could indicate sample warming or cooling during collection that could alter the carbonate system properties. No samples were flagged based on these parameters. Next, sample values were examined based on total alkalinity (TA)-salinity relationships determined from all samples collected on the same date (i.e., each cast), excluding low-salinity samples (S < 20) that were grouped together across all casts and examined separately. Low-salinity samples demonstrated a different TA/S relationship than higher-salinity samples with an inflection in the relationship at S = ~20 (see Fig. 4 in manuscript). TA and S regressions were produced for each sampling date (BI: n = 11 to 13, RI: n = 8 to 12) and measurements exceeding 3X (S ≥ 20) or 2X (S < 20) the root mean squared error (RMSE) (BI: S ≥ 20, 5 – 18 μmol kg^-1^, S < 20, 9 μmol kg^-1^, RI: S ≥ 20, 5 – 15 μmol kg^-1^, S < 20, 24 μmol kg^-1^) were initially flagged for further scrutiny. Flagged measurements were next inspected for accompanying independent measurements of oxygen and nutrients that confirmed the unusual TCO_2_ measurement. If the independent measurements were consistent with the measured TCO_2_ value, the flags were removed. For example, if an unusually high TCO_2_ measurement was accompanied by an unusually high oxygen and nutrient measurement, the flag was removed. Flagged samples remaining at the end of the QC process were excluded from further analyses.
