# Supplementary figures and images for: Contrasting marine carbonate systems in two fjords in British Columbia, Canada: Seawater buffering capacity and the response to anthropogenic CO2 invasion
Source: PLoS One. 2020 Sep 3;15(9):e0238432. doi: 10.1371/journal.pone.0238432 (PMC7470366; doi:10.1371/journal.pone.0238432)

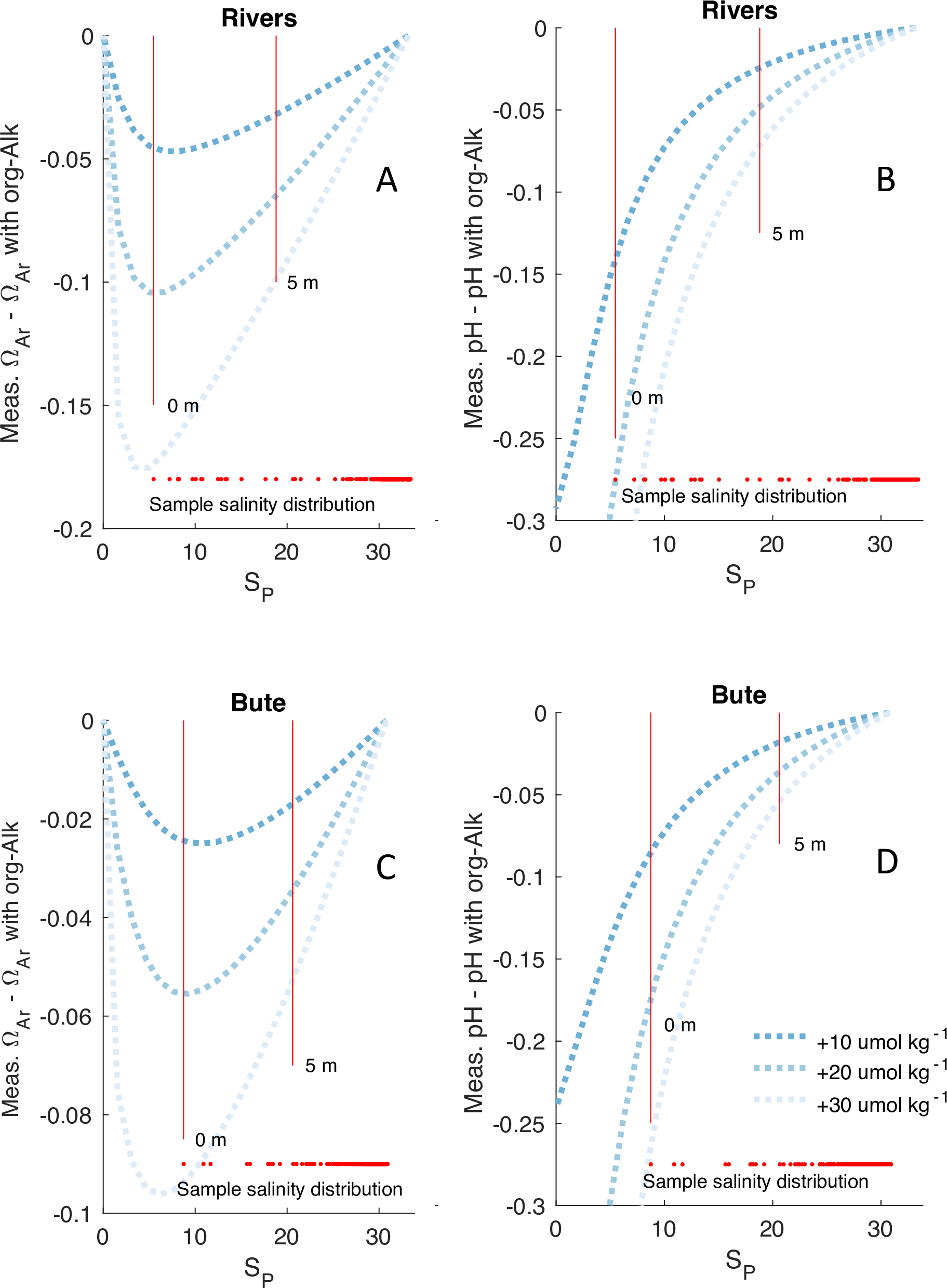

Supplement: S1 Fig — Blue dashed lines represent difference in ΩAr (panels A, C) and pH (panels B, D) computed from estimated TA and TCO2 relationships (S1 Table) plus organic alkalinity at concentrations indicated in figure legend, and without additional organic alkalinity. Legend in panel D applies to all panels. Red vertical lines indicate minimum salinity of 0 m and 5 m samples. Red circles indicate salinity distribution of all samples for the corresponding fjord. (TIF) [file pone.0238432.s002.tif]

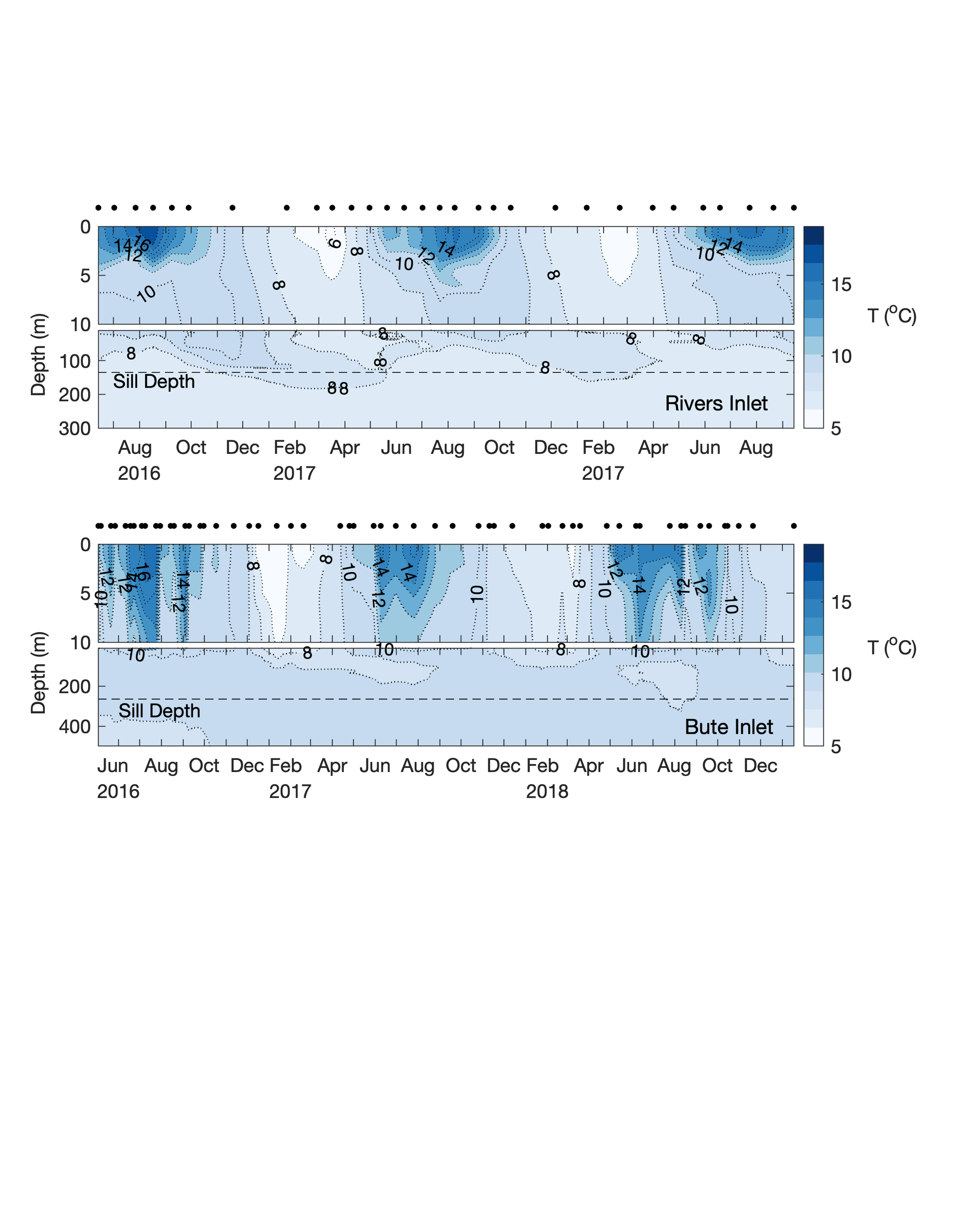

Supplement: S2 Fig — Black circles above panels indicate sampling dates. (TIF) [file pone.0238432.s003.tif]

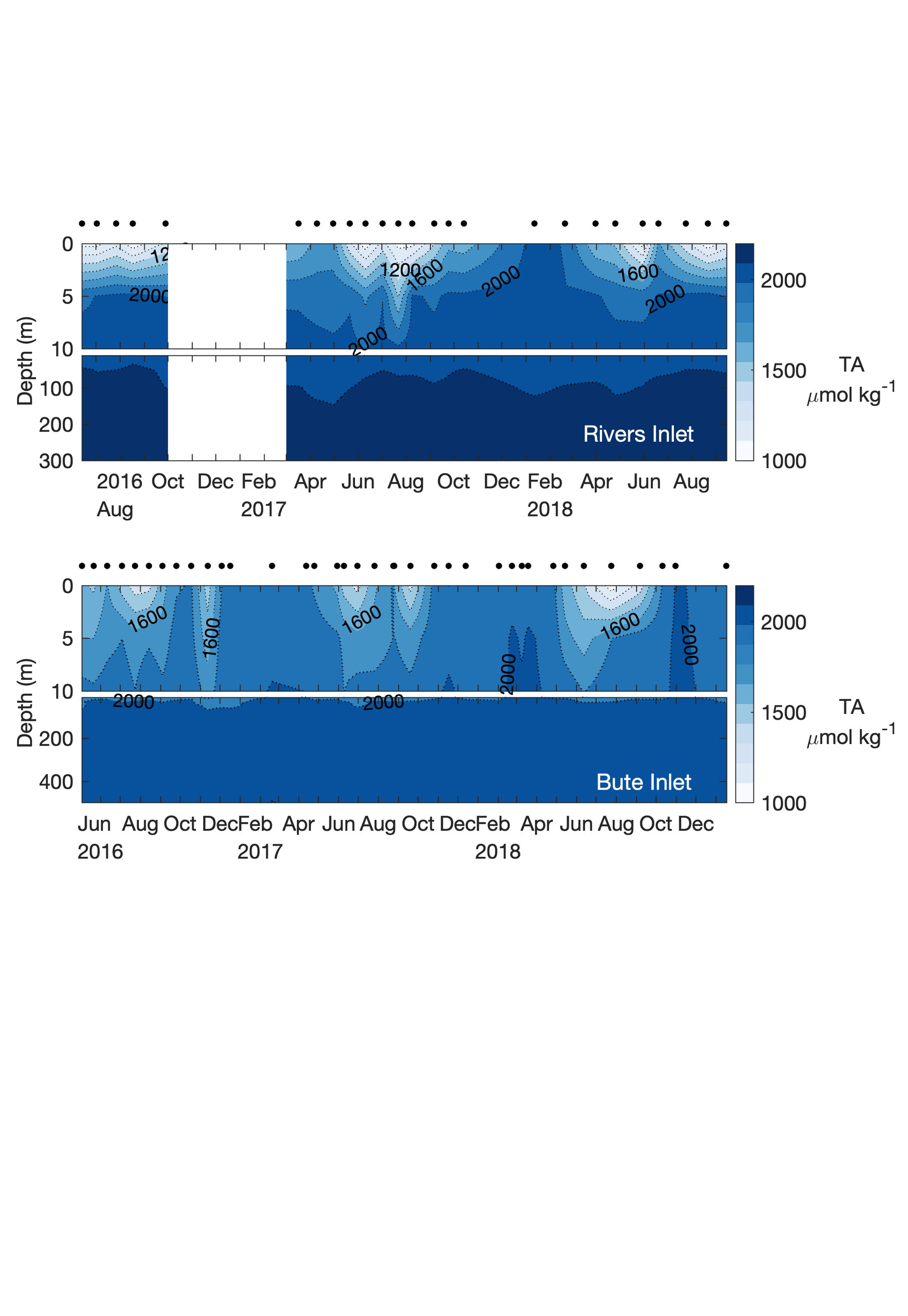

Supplement: S3 Fig — Black circles above panels indicate sampling dates. (TIF) [file pone.0238432.s004.tif]

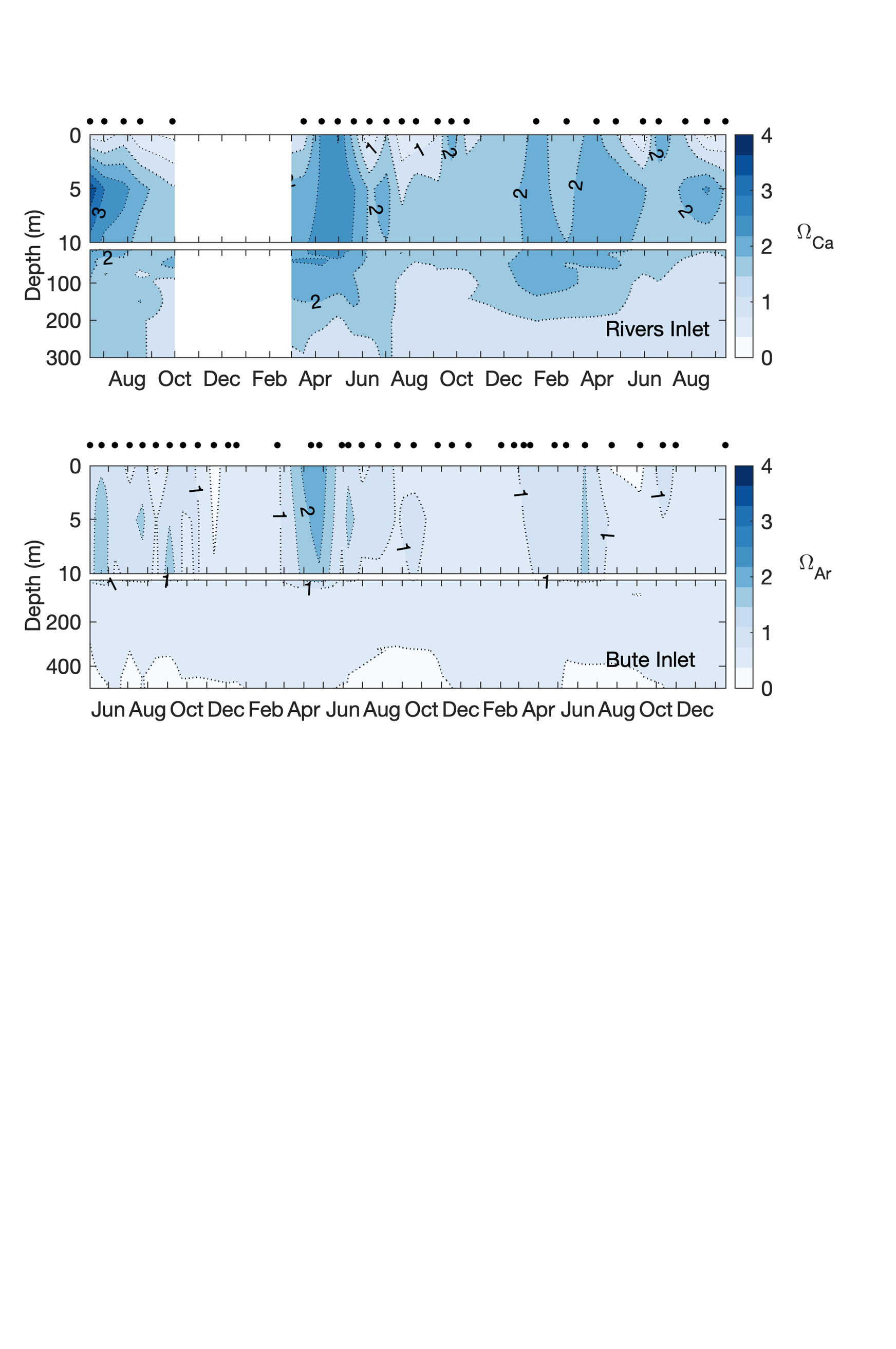

Supplement: S4 Fig — Black circles above plots indicate sampling dates. Contours of 1 in the lower ΩAr panel represent the saturation horizon. (TIF) [file pone.0238432.s005.tif]

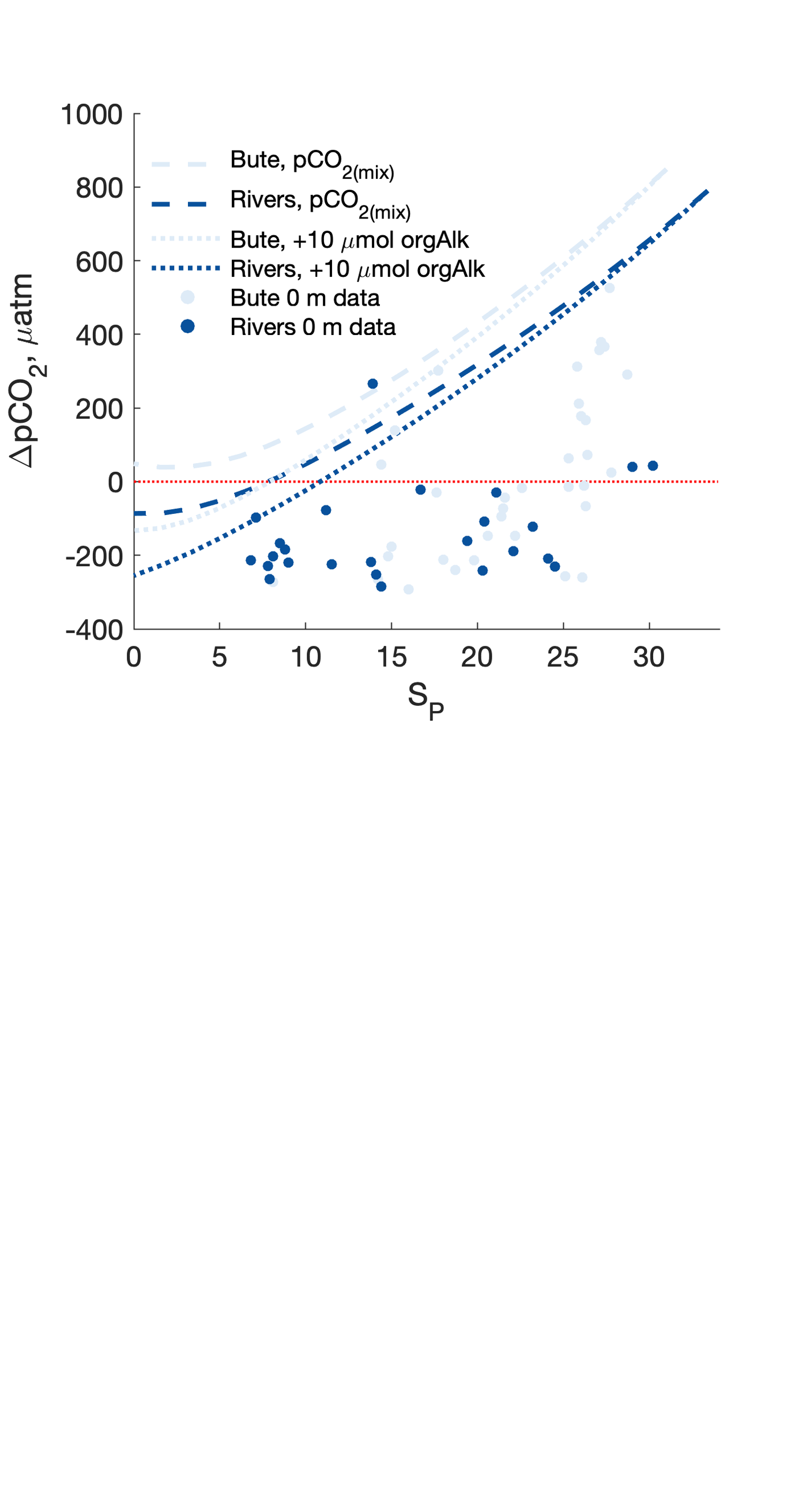

Supplement: S5 Fig — ΔpCO2 represents atmospheric pCO2 subtracted from the pCO2 corresponding to estimated freshwater and seawater TA and TCO2 concentrations determined from seawater relationships and river properties (S1 and S4 Tables) for mixing ratios from 0 to 1, represented by salinity of the mixture, without (dashed lines) and with (dotted lines) 10 μmol kg-1 organic alkalinity (orgAlk). ΔpCO2 is computed similarly from measured pCO2 for surface (i.e., 0 m) samples (filled circles). Red dotted line indicates atmospheric saturation. (TIF) [file pone.0238432.s006.tif]

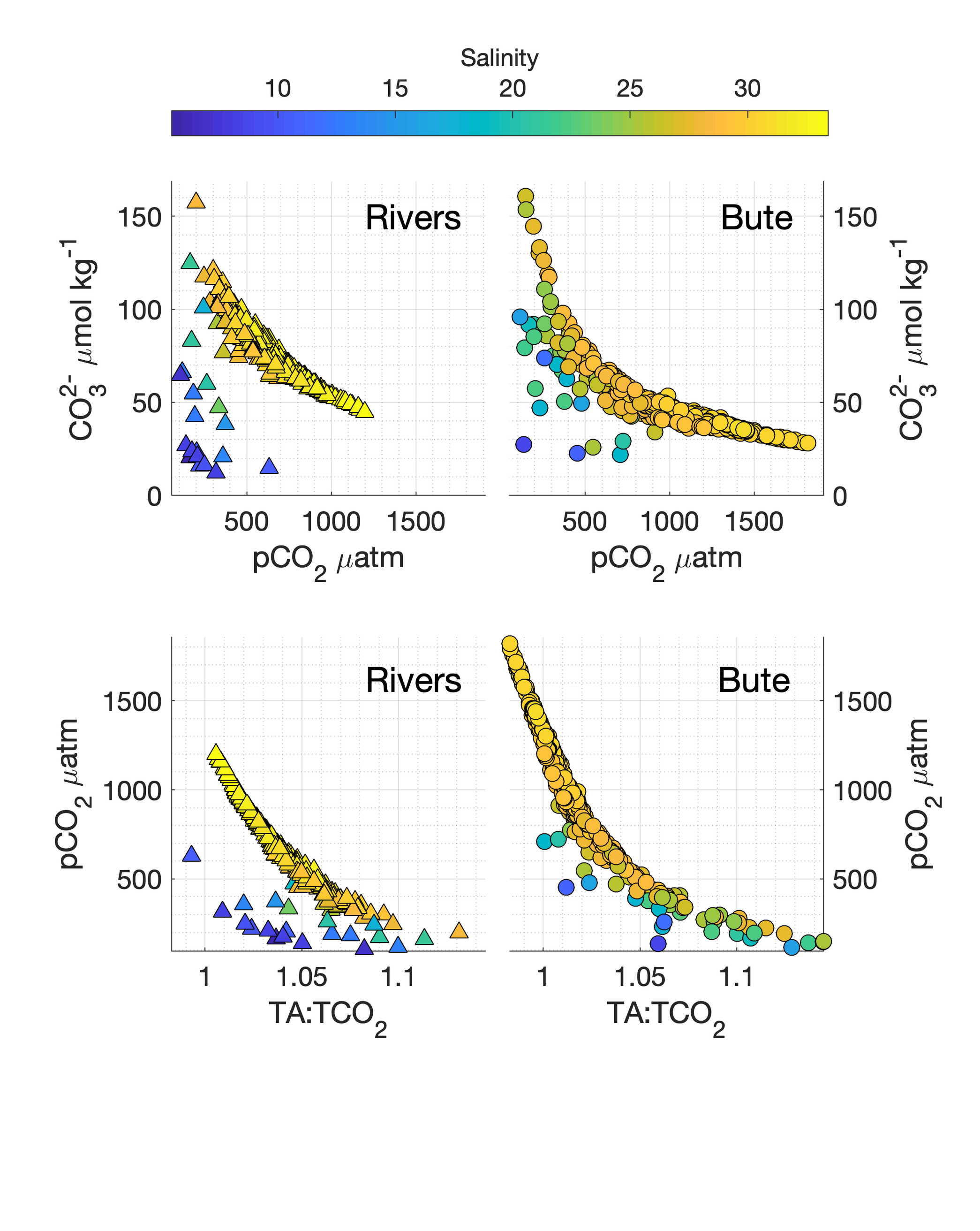

Supplement: S6 Fig — Colorbar applies to all panels. (TIF) [file pone.0238432.s007.tif]
