# Supplementary material for: Contrasting marine carbonate systems in two fjords in British Columbia, Canada: Seawater buffering capacity and the response to anthropogenic CO2 invasion
Source: PLoS One. 2020 Sep 3;15(9):e0238432. doi: 10.1371/journal.pone.0238432 (PMC7470366; doi:10.1371/journal.pone.0238432)
Supplement: S1 Table — (DOCX) [file pone.0238432.s008.docx]

**S1 Table.** Least-squares regression parameters for TCO_2_ and TA relationships with salinity in Bute and Rivers inlets.

| **Sample group** | **n** | **r^2^** | **m** | **b, μmol kg^-1^** | **rmse, μmol kg^-1^** |
| --- | --- | --- | --- | --- | --- |
| ***Bute Inlet***  TCO_2_, all samples | 415 | 0.973 | 71.2 | -79.4 | 34.6 |
| TCO_2_, S < 20 | 12 | 0.941 | 58.4 | 179.3 | 46.6 |
| TA, all samples | 415 | 0.990 | 62.9 | 191.3 | 18.1 |
| TA, S < 20 | 12 | 0.997 | 65.0 | 154.7 | 10.7 |
| ***Rivers Inlet***  TCO_2_, all samples | 266 | 0.986 | 61.5 | 131.1 | 38.5 |
| TCO_2_, S < 20 | 16 | 0.978 | 51.8 | 272.6 | 30.9 |
| TA, all samples | 266 | 0.996 | 62.1 | 193.8 | 20.8 |
| TA, S < 20 | 16 | 0.987 | 56.8 | 255.1 | 25.6 |
