# Supplementary material for: Contrasting marine carbonate systems in two fjords in British Columbia, Canada: Seawater buffering capacity and the response to anthropogenic CO2 invasion
Source: PLoS One. 2020 Sep 3;15(9):e0238432. doi: 10.1371/journal.pone.0238432 (PMC7470366; doi:10.1371/journal.pone.0238432)
Supplement: S2 Table — (DOCX) [file pone.0238432.s009.docx]

**S2 Table.** Mean Net Community Production (NCP) by month for the upper 30 m of the water column in Bute and Rivers inlets.

| **Fjord** | **Month** | **∆nTCO_2_^a^** | **TA_corr_^b^** | **NCP, μmol kg^-1^** | **mmol, C m^2^ d** |
| --- | --- | --- | --- | --- | --- |
| Bute Inlet | Jan | -11.6 ± 12.8^c^ | -3.6 ± 25.9 | -8 ± 28.9 | -8.2 ± 29.6 |
|  | Feb | 11.1 ± 12.9 | -10.3 ± 26.2 | 21.4 ± 29.2 | 21.9 ± 29.9 |
|  | Mar | 49.1 ± 13.4 | 11.9 ± 25.7 | 37.1 ± 29.0 | 38 ± 29.6 |
|  | Apr | 36.2 ± 17.9 | -12.5 ± 25.6 | 48.7 ± 31.2 | 49.8 ± 31.9 |
|  | May | -39.9 ± 16.6 | 10.5 ± 25.6 | -50.3 ± 30.5 | -51.4 ± 31.2 |
|  | Jun | -11.5 ± 16.4 | -7.7 ± 25.6 | -3.8 ± 30.4 | -3.9 ± 31.0 |
|  | Jul | -6.2 ± 22.0 | 9 ± 25.7 | -15.2 ± 33.8 | -15.5 ± 34.5 |
|  | Aug | -23.4 ± 21.6 | 2.2 ± 25.6 | -25.6 ± 33.5 | -26.1 ± 34.2 |
|  | Sep | 23.5 ± 17.2 | 5.1 ± 25.5 | 18.4 ± 30.7 | 18.8 ± 31.3 |
|  | Oct | -0.5 ± 15.0 | -4.8 ± 25.3 | -4.3 ± 29.5 | -4.4 ± 30.1 |
|  | Nov | -30.1 ± 16.8 | -1.5 ± 25.9 | -28.7 ± 30.9 | -29.3 ± 31.6 |
|  | Dec | 3.4 ± 15.4 | 1.7 ± 25.9 | 1.7 ± 30.2 | -1.7 ± 30.8 |
|  |  |  |  |  |  |
| Rivers Inlet | Jan | n.d.^d^ | n.d. | n.d. | n.d. |
|  | Feb | 12.5 ± 11.5 | 10.8 ± 25.3 | 1.7 ± 27.8 | 1.7 ± 28.4 |
|  | Mar | 4.4 ±15.4 | -6.7 ± 26.1 | 11.1 ± 30.3 | 11.3 ± 30.9 |
|  | Apr | 38.0 ± 21.6 | 6.7 ± 26.4 | 31.3 ± 34.2 | 32.0 ± 34.9 |
|  | May | -62.0 ± 27.9 | 1.6 ± 26.0 | -63.7 ± 38.1 | -65.0 ± 38.9 |
|  | Jun | -4.6 ± 26.7 | -8.4 ± 26.4 | 3.8 ± 37.4 | 3.9 ± 38.2 |
|  | Jul | -0.0 ± 20.5 | -6.1 26.0 | 6.0 ± 33.1 | 6.1 ± 33.8 |
|  | Aug | -35.7 ± 38.5 | 1.5 ± 29.7 | -37.2 ± 48.6 | -38.0 ± 49.6 |
|  | Sep | 32.8 ± 36.7 | 18.3 ± 29.7 | 14.5 ± 47.2 | 14.8 ± 48.2 |
|  | Oct | -4.15 ± 12.2 | -6.6 ± 25.0 | 2.3 ± 27.9 | 2.4 ± 28.5 |
|  | Nov | n.d. | n.d. | n.d. | n.d. |
|  | Dec | n.d. | n.d. | n.d. | n.d. |

^a^ Change in nTCO_2_ between the mean nTCO_2_ of the corresponding and previous months.

^b^ Correction factor accounting for differences in mean monthly TA and NO_3_ concentrations, computed according to the equation ∆nTA + ∆NO_3_^-^ × 0.5. See Methods for complete equations.

^c^ Uncertainties represent the propagation of standard error of the mean monthly concentrations plus analytical uncertainties.

^d^ n.d. = no data available.
