# Supplementary material for: Contrasting marine carbonate systems in two fjords in British Columbia, Canada: Seawater buffering capacity and the response to anthropogenic CO2 invasion
Source: PLoS One. 2020 Sep 3;15(9):e0238432. doi: 10.1371/journal.pone.0238432 (PMC7470366; doi:10.1371/journal.pone.0238432)
Supplement: S3 Table — (DOCX) [file pone.0238432.s010.docx]

**S3 Table.** Selected output conditions of the ∆TCO_2_ method under pCO_2_ trajectories associated with the RCP 8.5 and RCP 6.0 scenarios.

| **Fjord** | **Condition** | **RCP 8.5** | **RCP 6.0** |
| --- | --- | --- | --- |
| Rivers Inlet | TCO_2_Anth,_ μmol kg^-1^, 2017 surface layer | 50 ± 4 | 48 ± 3 |
|  | TCO_2_Anth_ μmol kg^-1^, 2017 deep layer, | 34 ± 3 | 34 ± 3 |
|  | First year mean surface layer Ω_Ar_ < 1 | 2057 ± 8 | 2073 ± 11 |
|  |  |  |  |
| Bute Inlet | TCO_2_Anth_ μmol kg^-1^, 2017 surface layer, | 47 ± 3 | 46 ± 3 |
|  | TCO_2_Anth_ μmol kg^-1^, 2017 deep layer, | 32 ± 2 | 32 ± 2 |
|  | First year mean surface layer Ω_Ar_ < 1 | 2055 ± 8 | 2075 ± 11 |
