# Supplementary material for: Contrasting marine carbonate systems in two fjords in British Columbia, Canada: Seawater buffering capacity and the response to anthropogenic CO2 invasion
Source: PLoS One. 2020 Sep 3;15(9):e0238432. doi: 10.1371/journal.pone.0238432 (PMC7470366; doi:10.1371/journal.pone.0238432)
Supplement: S4 Table — (DOCX) [file pone.0238432.s011.docx]

**S4 Table.** Mean water temperature in the Homathko and Wannock rivers between May and August for the years 2017 to 2019.

| **River** | **Mean**  **Temperature**^a^ **(∘C)** | **[Ca^2+^]**  **(μM)** |
| --- | --- | --- |
| Homathko River (Bute Inlet) | 8.2 | 70 to 398^b, c^ |
| Wannock River (Rivers Inlet) | 11.6 | 93 to 109^c^ |

^a^ Data from [1].

^b^ Data from [2].

^c^ Data from [3].

References cited:

1. Water Survey of Canada. Historical Hydrometric Data Search: Government of Canada; 2019 [updated 2019-08-05; cited 2019 09-04]. Available from: [https://wateroffice.ec.gc.ca/search/historical_e.html](about:blank).
2. Kirsch, M. Ionic Ratios of Some of the Major Components in River-Diluted Sea Water in Bute and Knight Inlets, British Columbia. J. Fish. Res. Bd. Canada, 13(3), pp. 273–289, 1956
3. Giesbrecht, I. (March 13, 2020) Email communication.
