# Supplementary material for: Contrasting marine carbonate systems in two fjords in British Columbia, Canada: Seawater buffering capacity and the response to anthropogenic CO2 invasion
Source: PLoS One. 2020 Sep 3;15(9):e0238432. doi: 10.1371/journal.pone.0238432 (PMC7470366; doi:10.1371/journal.pone.0238432)
Supplement: S5 Table — (DOCX) [file pone.0238432.s012.docx]

**S5 Table.** Bottom water properties used to estimate deep water respiration in Rivers and Bute inlets.

|  | **Rivers Inlet^a^** | **Bute Inlet^b^** | **Continental Shelf^c^** | **Southern**  **Salish Sea^d^** | **Northern Salish Sea^e^** |
| --- | --- | --- | --- | --- | --- |
| **Depth (m)** | 300 | 500 | 130 | ~ 225 | 260 |
| **Salinity** | 33.3 | 30.8 | 33.7 | 33.8 | 30.8 |
| **TCO_2_ (μmol kg^-1^)** | 2220 | 2157 | 2224 | 2200 | 2090 |

^a^ Mean bottom water conditions for April and May

^b^ Mean bottom water conditions for July and August

^c^ Reference [67] in manuscript

^d^ Reference [59] in manuscript

^e^ Reference [38] in manuscript
